# Evolution of terpene synthases in the sesquiterpene biosynthesis pathway and analysis of their transcriptional regulatory network in Asteraceae

**DOI:** 10.1093/hr/uhaf229

**Published:** 2025-09-02

**Authors:** Xiuping Yang, Fanbo Meng, Qian Cheng, Pengmian Feng, Xiaoming Song, Wei Chen

**Affiliations:** School of Pharmacy, Chengdu University of Traditional Chinese Medicine, Chengdu 611137, China; School of Basic Medicine, Chengdu University of Traditional Chinese Medicine, Chengdu 611137, China; Innovative Institute of Chengdu University of Traditional Chinese Medicine, Chengdu University of Traditional Chinese Medicine, Chengdu 611137, China; School of Pharmacy, Chengdu University of Traditional Chinese Medicine, Chengdu 611137, China; School of Basic Medicine, Chengdu University of Traditional Chinese Medicine, Chengdu 611137, China; School of Basic Medical Sciences, North China University of Science and Technology, Tangshan 063210, China; School of Pharmacy, Chengdu University of Traditional Chinese Medicine, Chengdu 611137, China; School of Basic Medicine, Chengdu University of Traditional Chinese Medicine, Chengdu 611137, China; Innovative Institute of Chengdu University of Traditional Chinese Medicine, Chengdu University of Traditional Chinese Medicine, Chengdu 611137, China

## Abstract

The Asteraceae family, one of the largest angiosperm families, is rich in terpenoid secondary metabolites with significant medicinal value. Asteraceae plants have evolved a diverse array of terpenoid biosynthesis pathways, reflecting their adaptive significance and complex regulatory mechanisms. However, the evolutionary patterns and transcriptional regulatory mechanisms governing these biosynthetic processes remain unclear. This study investigates the evolution and transcriptional regulation of terpenoid biosynthesis genes in Asteraceae. Comparative genomic analysis of 19 Asteraceae and six out-group species revealed that Asteraceae species diverged ~74.03 million years ago and were distinctly divided into three subfamilies. A total of 1714 terpene synthase (TPS) genes were identified, predominantly in the TPS-a and TPS-b subfamilies. Caryophyllene-type sesquiterpene biosynthetic gene clusters (BGCs) were detected in 10 species, with their formation due to whole-genome duplication (WGD) and tandem duplication. By integrating weighted gene coexpression network analysis (WGCNA) and machine learning methods, key transcription factors regulating caryophyllene synthase (CPS) in *Carthamus tinctorius* were identified. A multilayered gene regulatory network was constructed to identify potential regulatory factors involved in TPS gene regulation under light stress. By exploring the evolutionary patterns and potential regulatory relationships involved in terpenoid biosynthesis in Asteraceae, this study provides important insights into TPS gene evolution. In addition, the findings also offer guidance for optimizing genetic engineering strategies in terpenoid-based drug development.

## Introduction

Plant secondary metabolites have a dual role in responses to biotic and abiotic stresses [1]. They not only participate in ecological defense but also serve as important sources of pharmaceutically active ingredients. Among them, terpenoids, one of the most diverse types of secondary metabolites, are synthesized through the mevalonate (MVA) and 2-C-methyl-D-erythritol 4-phosphate (MEP) pathways, encompassing various subtypes, such as monoterpenoids, sesquiterpenoids, and diterpenoids [2, 3]. Sesquiterpenoids, which contain three isoprene units, are widely distributed in plants [4] and can be classified into subtypes like germacrane [5] and caryophyllane [6] based on their carbon skeletons. Sesquiterpenoid compounds exhibit a dual functionality: on one hand, they participate in plant immune responses to resist biotic stresses and alleviate oxidative damage [7–9]; on the other hand, they exhibit pharmacological activities such as antioxidant, anti-inflammatory, and antitumor effects, making them highly promising for drug research, development, and clinical applications [10].

The Asteraceae family is one of the largest families of seed plants found worldwide [11, 12] and has ornamental, economic, and medicinal values [13]. Medicinal plants in the Asteraceae family are rich in active ingredients, with terpenoids being particularly prominent and receiving extensive research attention [13]. The sesquiterpenoids present in these plants include guaianolide sesquiterpenoids [14], artemisinin [15, 16], sesquiterpene lactones [17], caryophyllene-type sesquiterpenoids [18], etc. Caryophyllene-type sesquiterpenoids have attracted much attention due to their pharmacological activities such as antidiabetes, hepatoprotection, and neuroprotection [19]. In Asteraceae species, caryophyllene sesquiterpenoids are one of the important pharmacologically active components [20]. Caryophyllene in chrysanthemum essential oil can exert cytotoxic effects by inducing cell cycle arrest in lung cancer cells [21]. Additionally, caryophyllene sesquiterpenoids in *Carthamus tinctorius* exhibit promising potential for antiatherosclerosis activity [18].

Gene clusters have long been recognized in metabolic pathways of fungi and bacteria [22, 23]. With the advent of high-throughput sequencing, high-quality genomes of an increasing number of plants have been completed, leading to the discovery of numerous biosynthetic gene clusters [24]. To date, genome sequences of ~46 different Asteraceae species have been published. These include *Artemisia annua* [25], *Artemisia argyi* [26], *Lactuca sativa* [27], *Helianthus annuus* [28], *Chrysanthemum morifolium* Ramat [29], *C. tinctorius* [30], and so on. However, studies on the biosynthetic gene clusters of terpenoids in the Asteraceae family remain limited, hindering our understanding of the formation and function of these gene clusters. Biosynthetic genes typically form through the physical aggregation of functionally related nonhomologous genes [7, 31, 32]. So far, >30 plant biosynthetic gene clusters (BGCs) have been functionally verified, present in compounds such as the miltiradiene BGCs in the Lamiaceae family [32], the benzylisoquinoline alkaloids gene cluster in poppies [33], and the two BGCs involved in the synthesis of hypericin in *Hypericum perforatum* [34]. However, the origin and evolution of BGCs remain unclear. Some studies suggest that their formation may involve chromosomal remodeling, transposition, or gene duplication events [35, 36].

Transcription factors (TFs) are a class of proteins that regulate the expression of specific genes by binding to DNA, among which MYB family play a particularly important role in regulating terpenoid synthesis [37]. In *Salvia miltiorrhiza*, *SmMYB9b* significantly upregulates the expression of key terpenoid synthesis genes such as *DXS*, *GGPPS*, and *KSL1*. Additionally, overexpression of *SmMYB97* and *SmMYB98* has been shown to markedly increase the accumulation of tanshinones [38]. In *A. annua*, MYB such as *AaTAR2* and *AaMYB17* positively regulate trichome development, leading to enhanced artemisinin production [39]. These findings demonstrate that MYB exert positive regulatory effects on the biosynthesis of pharmacologically active compounds in various medicinal plants. Understanding these regulatory mechanisms provides a theoretical foundation and technical framework for molecular breeding aimed at developing high-yield plant varieties with elevated levels of medicinal terpenoids.

In this study, tools such as OrthoFinder [40], MCMCtree [41–43], TimeTree [44], and MCScan [45] were used to reconstruct the overall evolutionary relationships of the Asteraceae family and the evolutionary patterns of terpene synthase (TPS) genes across 19 Asteraceae species. By analyzing the genomic data of these species, we investigated the evolution of terpenoid biosynthesis pathways and the corresponding terpene BGCs in the Asteraceae family. Additionally, by integrating transcriptome data, we used a computational inference algorithm to construct a multilayered gene regulatory network (GRN) of *C. tinctorius* under light stress. This analysis enabled the identification of TFs potentially involved in the regulation of TPS genes. Our findings not only provide new insights into the genetic basis of the terpenoid metabolic diversity in Asteraceae species, but also offer valuable implications for understanding their adaptive responses to environmental stimuli.

## Results

### Phylogenetic analysis of Asteraceae

**Figure 1:**
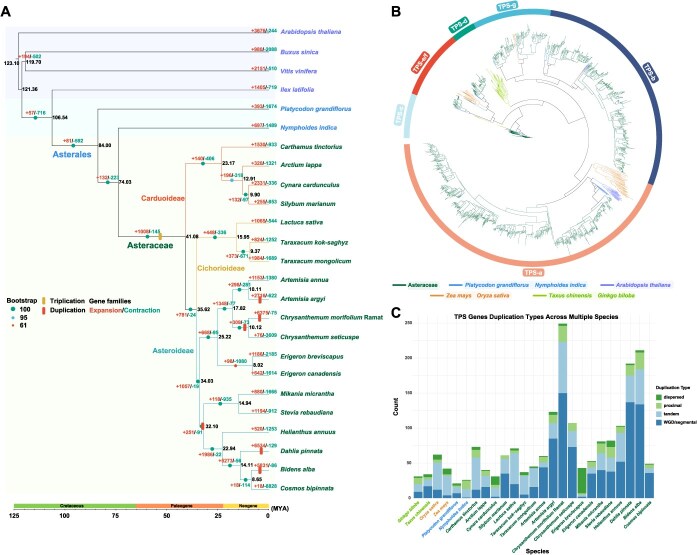
Phylogenetic analysis of Asteraceae species and the TPS gene family. (A) Phylogenetic tree of 19 Asteraceae species along with six outgroup species. Dots indicate bootstrap of the evolutionary tree. Bars of different colors indicate whole-genome triplication and duplication events, respectively. Gene-family expansion and contraction events are highlighted in different colors in the figure. (B) Phylogenetic tree of TPS genes from 19 Asteraceae species and 6 outgroup species. Branches of different colors represent different taxonomic groups of species. (C) The duplication patterns of TPS genes identified in 25 species.

To construct a species relationship framework for the evolution of sesquiterpene biosynthetic gene clusters in Asteraceae, 19 Asteraceae species along with six outgroup species were selected. Using OrthoFinder (v2.5.4), we identified the orthologous gene families and screened out 107 low-copy orthologous gene families (OGs). After rigorous selection, we obtained a high-quality low-copy gene and constructed a concatenated dataset of amino acids together with corresponding coding sequences. The phylogenetic tree was built using the FastTree (Fig. 1A). The results showed that the Asteraceae exhibited a clear branching structure on the phylogenetic tree. The species were clearly divided into three subfamilies: Carduoideae, Cichorioideae, and Asteroideae. Evolutionary analysis suggested that Asteraceae experienced a shared whole-genome triplication (WGT) event, with an additional genome duplication occurring specially within the Asteroideae subfamily. The phylogenetic tree demonstrated strong branch support, with most branches achieving a support rate of 100%. However, the branches of *Erigeron breviscapus* and *Erigeron canadensis* had a support rate of 61%, while *Arctium lappa* and *Silybum marianum* had a support rate of 95%. Molecular clock analysis suggested that Asteraceae diverged from other families ~74.03 million years ago (MYA), Carduoideae diverged from the clade comprising Cichorioideae and Asteroideae ~41.08 MYA, and Cichorioideae and Asteroideae separated ~35.62 MYA. These findings will provide valuable insights into the evolutionary history of Asteraceae.

Analysis of gene family expansion and contraction revealed that the Asteraceae subfamily species *C. morifolium*, *Dahlia pinnata*, and *Bidens alba* underwent a recent duplication event after diverging from their sister species. Compared with these sister species, their gene families expanded on a large scale (Fig. 1A). The expanded gene families of *C. morifolium* were mainly associated with biological processes including regulation of transport, vesicle-mediated transport, positive regulation of cellular communication, and organic acid transport (Fig. S1, Table S1), which are crucial for plant growth and development, as well as responses to biotic and abiotic stresses. In *D. pinnata*, the expanded gene families were mainly enriched in biological processes related to cell death, response to red or far-red light, and sulfur compound metabolic processes. In *B. alba*, the enriched processes among expanded gene families included xenobiotic transmembrane transport, response to fungal infection, and cell development (Fig. S2, Tables S2 and S3). These findings suggest that the expanded gene families in *D. pinnata* and *B. alba* are functionally linked to metabolic regulation, stress responses, and developmental processes, which are likely to play crucial roles in the growth, development, and environmental adaptation of these species.

In addition, gene ontology (GO) enrichment analysis of the 1008 expanded gene families at the divergence nodes between Asteraceae and outgroup species revealed that these gene families were mainly enriched in biological processes such as regulation of postembryonic root development, negative regulation of ethylene-activated signaling pathway, negative regulation of phosphorelay signal transduction system, microtubule nucleation, and cell adhesion. These processes were involved in growth and development, morphological construction optimization, environmental adaptation, and stress response (Fig. S3, Table S4). The dynamic changes in these gene families reflect the functional and numerical adjustments of genes during the evolution of Asteraceae, which might be closely related to the evolution of plant morphology, ecological adaptability, and physiological traits.

### Identification and analysis of TPS gene family in Asteraceae species

To explore the evolutionary patterns of the TPS gene family in Asteraceae species, TPS genes were screeded in 19 Asteraceae species using two Hidden Markov Model (HMM) profiles (PF01397, PF03936) [46]. A total of 1714 TPS genes were identified (Fig. 1B). Among them, the TPS-a and TPS-b subfamilies contained the largest number of genes and were mainly involved in the synthesis of sesquiterpenes and monoterpenes. Compared with close relatives *Platycodon grandiflorus* and *Nymphoides indica*, the TPS-a and TPS-b subfamilies in Asteraceae species underwent gene amplification via tandem duplication and segmental duplication. Most Asteraceae species possess a higher number of TPS genes than gymnosperms and monocots, suggesting that the TPS gene family may have undergone lineage-specific expansion during the evolutionary divergence of Asteraceae from other plant families (Fig. 1C, Fig. S4, Table S5). In nine Asteraceae species, most TPS genes expanded through tandem and segmental duplications are expressed in various tissues such as roots, stems, leaves, and flowers, indicating their involvement in terpene biosynthesis and potential contribution to increased terpene accumulation (Figs S5 and S6). These findings highlight the crucial role of TPS-a and TPS-b subfamilies in terpenoid biosynthesis in Asteraceae species, and are important for plant growth and development, defense responses, and environmental adaptability.

**Figure 2:**
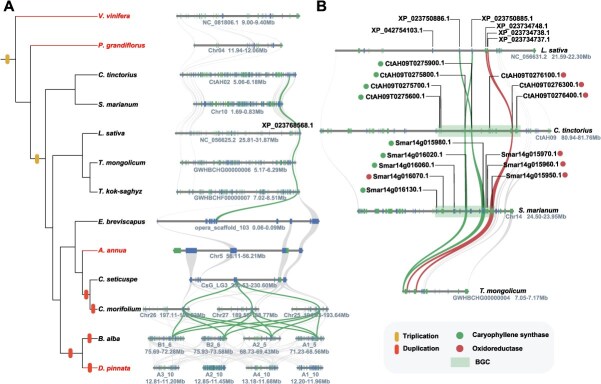
Evolution of Caryophyllene Synthase genes in Asteraceae. (A) Microsynteny analysis of CPS regions across 13 species. Rectangles represent annotated genes, with genes on the reverse strand and forward strand distinguished by different colors of the rectangles. The names of CPS genes in *L. sativa* are labeled above the corresponding gene blocks; syntenic genes are connected by curves, and syntenic CPS genes are identified by highlighting. The phylogenetic tree on the left side of the figure shows the evolutionary relationships among 13 species; the differently colored bands in the figure correspond to whole-genome triplication events and gene duplication events, respectively. Species with highlighted names represent those for which no homologous genes were detected. (B) Microsynteny analysis of CPS regions in four Asteraceae species. The six genes in *L. sativa* are labeled above the gene blocks. Syntenic CPS genes and oxidoreductase genes are highlighted with curves of different colors, respectively. Square regions in the figure represent BGCs; genes within the clusters are labeled above the gene blocks, and distinguished by different colors according to their functional differences.

By comparing the relative abundances of TPS genes in the gene pools of species (Table S5), it was found that *C. tinctorius* had the highest relative abundance of TPS genes. Analysis of the formation mechanisms of 73 TPS genes in *C. tinctorius* revealed that 46 were primarily generated through tandem duplication, and 12 originated from segmental duplication. Among the remaining 15 TPS genes, four were identified as dispersed duplication, while the other 11 were categorized as proximal duplication (Fig. S7). According to the distribution of *C. tinctorius* terpene synthase (*CtTPS*) genes on chromosomes, *CtTPS* formed a large gene cluster through tandem duplication on chromosome 11. With the exception of chromosomes 7, 8, and 9, tandem duplication events were detected on all other chromosomes (Fig. S7, Table S6). This gene expansion may explain the high TPS gene abundance in *C. tinctorius*. The high abundance of TPS genes provides a genetic basis for the synthesis of diverse terpenoids, which may confer a competitive advantage to *C. tinctorius* in its ecological environment.

In addition, the relative abundances of TPS genes in *A. argyi*, *C. morifolium*, *Mikania micrantha*, and *Stevia rebaudiana* were also higher than those in other species (Fig. 1B, Fig. S4, and Table S5). Although the species in the subfamily Asteroideae have undergone additional whole-genome duplication (WGD) events, their TPS genes did not show a consistent increasing trend compared to the other two subfamilies. This phenomenon suggests that the evolution of the TPS gene family in Asteraceae may be influenced by multiple complex regulatory factors.

### Insights into the formation and evolution of terpene BGCs in Asteraceae species

Caryophyllene sesquiterpenoids are bioactive components in Asteraceae species. To investigate their biosynthetic pathways, a systematic identification of the terpene BGCs was conducted in 19 Asteraceae species using the PlantiSMASH tool. *Chrysanthemum morifolium* had the largest number of terpene BGCs (*n* = 39), followed by *B. alba* (*n* = 32, Table S7). Notably, no terpene BGCs were detected in *Cynara cardunculus*. To elucidate the functions of the TPS genes within these gene clusters, functionally verified TPS genes of *L. sativa*, *C. cardunculus*, and *H. annuus* were retrieved from the NCBI database. Through sequence alignment and phylogenetic analysis, the TPS genes of Asteraceae species were classified into nine functional evolutionary branches associated with sesquiterpene biosynthesis (Fig. S8). Among the species containing sesquiterpene BGCs, caryophyllene-type sesquiterpene BGCs were identified in 10 Asteraceae species, including *C. tinctorius*, *S. marianum*, *C. morifolium*, *D. pinnata*, *B. alba*, *Chrysanthemum seticuspe*, *A. annua*, *E. breviscapus*, *Taraxacum mongolicum*, and *Taraxacum kok-saghyz*. Among them, *C. morifolium* had the highest number of such BGCs (*n* = 9, Fig. S9).

The synteny relationships within the *C. morifolium* were analyzed using MCScan. The results showed that there was collinearity between BGC2 and BGC3, between BGC5 and BGC6, and among BGC7, BGC8, and BGC9 in *C. morifolium* (Fig. S10). These findings suggest that the formation of these BGCs is closely related to WGD that occurred in *C. morifolium*. Moreover, gene loss was observed in some chromosomes during the duplication process, resulting in BGCs being present on only two out of three homologous chromosomes (Fig. S11). Remarkably, species that have undergone multiple WGD events exhibited significant expansion of BGCs, suggesting that WGD events have played a key role in the diversification of terpene biosynthesis, thereby enhancing ecological adaptability.

To trace the evolutionary trajectory of pharmacologically valuable caryophyllene-type sesquiterpenoids, a synteny analysis was conducted across 10 Asteraceae species to explore the evolution of caryophyllene synthase (CPS). CPS homologous to the XP_023768568.1 in *L. sativa* was found to be widely distributed in Asteraceae species, except in *A. annua* and *D. pinnata* (Fig. 2A). This suggests that CPS already existed in the common ancestor of Asteraceae, with gene loss occurring in certain species over the evolutionary process. Homologs of XP_023750886.1 and XP_023750885.1 were only found in *C. tinctorius*, *S. marianum*, and *T. mongolicum*. Interestingly, BGC was found in the homologous regions of *C. tinctorius* and *S. marianum*, and oxidoreductases within this BGC exhibited syntenic relationships among *L. sativa*, *C. tinctorius*, *S. marianum*, and *T. mongolicum*. However, according to the PlantiSMASH analyses, no BGCs were detected in this region in *L. sativa* and *T. mongolicum*. Comparative analysis revealed that BGCs in *C. tinctorius* and *S. marianum* contained a higher number of CPS and oxidoreductases (Fig. 2B), which were clustered through tandem duplication, contributing to the formation of gene clusters (Table S8). This suggests that BGC formation is not solely driven by WGD events but also by tandem duplication of functional genes. Through synteny analysis, no direct linear relationships were found among the caryophyllene-type sesquiterpene BGCs of other species. However, sequence alignment demonstrated high similarity and consistency among the CPS genes in these BGCs (Table S9), suggesting that these genes are likely homologous and that syntenic regions may have been lost during species divergence.

Although no BGCs for caryophyllene sesquiterpene synthesis were identified in *L. sativa*, a terpene BGC associated with the biosynthesis of germacrene A acid was discovered (Fig. S12). Within this gene cluster, two germacrene A synthase (GAS) genes exhibited high expression levels in roots, stems, and leaves, whereas germacrene A oxidase (GAO) was only expressed at low levels in stems and roots. It is worth noting that the CoA ligase (XP_042753758.1) within this cluster was not expressed in any of these tissues.

### Expression profiles and synteny analysis of genes in the terpene synthesis pathway of Asteraceae

**Figure 3:**
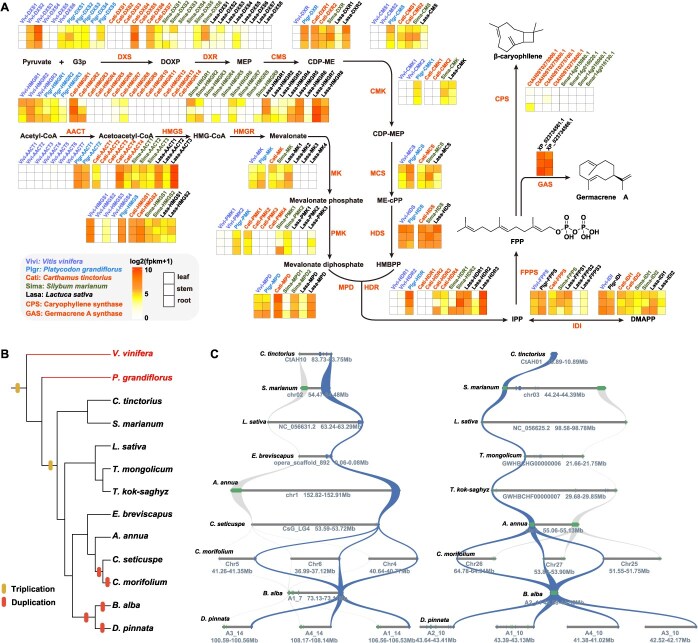
Analysis of gene expression profiles and synteny of sesquiterpenoid biosynthesis pathways in Asteraceae and outgroup species. (A) An overview of the sesquiterpenoid biosynthetic pathway, along with a heat map illustrating the expression profiles of candidate genes in *V. vinifera* (Vivi), *P. grandiflorus* (Plgr), *L. sativa* (Lasa), *C. tinctorius* (Cati), and *S. marianum* (Sima). (B) The evolutionary relationships among 13 species are shown, and the differently colored bands in the figure correspond to whole-genome triplication events and gene duplication events, respectively. Species with highlighted names represent those for which no homologous genes were detected. (C) Microsynteny analysis of the *HMGR* gene regions across 13 species. Rectangles represent annotated genes, with genes on the reverse strand and forward strand distinguished by different rectangle colors; syntenic genes are connected by curves, and syntenic *HMGR* genes are identified by highlighting.

Homologous caryophyllene-type sesquiterpene BGCs were identified in *C. tinctorius* and *S. marianum*. Given that these two species diverged relatively early in the evolutionary history of Asteraceae, the presence of conserved BGCs suggests a deeper evolutionary association between them. To better understand the evolutionary origins and conservation of these BGCs, comparative analyses were conducted across multiple species. *Vitis vinifera* and *P. grandiflorus* were selected as outgroup comparison species, while *L. sativa* was chosen as the ingroup comparison species. By analyzing the terpenoid biosynthetic pathways in *S. marianum* and *C. tinctorius*, and comparing gene collinearity across 10 species harboring caryophyllene-type sesquiterpene BGCs, we explored the evolutionary patterns of terpenoid biosynthesis in Asteraceae. The results revealed that genes in the terpenoid biosynthetic pathways of *V. vinifera* and *P. grandiflorus, L. sativa*, *C. tinctorius*, were highly expressed in roots, stems, and leaves. Moreover, these genes exhibited similar tissue-specific expression profiles (Fig. 3A, Table S10). Notably, the CPS gene in the gene cluster of *C. tinctorius* showed predominant expression in roots, with moderate expression in stems and leaves. In contrast, the CPS gene in the BGC of *S. marianum* exhibited extremely low expression or undetectable expression in all three tissues (Fig. 3A, Table S11). These findings suggest that the BGC in *C. tinctorius* may play an active role in regulating caryophyllene sesquiterpenoid biosynthesis, whereas the function of the corresponding BGC in *S. marianum* may have weakened or diverged over evolutionary time.

To investigate the evolution of the terpene synthesis pathway within Asteraceae, *V. vinifera*, *P. grandiflorus*, and *L. sativa* were used as a reference species for synteny analysis across 10 other Asteraceae species. Among the upstream genes, *HMGR* exhibited the highest level of conservation with a linear syntenic relationship observed across all 11 Asteraceae species (Fig. 3C). Similarly, *DXR* and *DXS* showed relatively high conservation, maintaining synteny across all Asteraceae species except *E. breviscapus* (Figs S13 and S14). Other upstream genes, including *ATOT*, *FPPS*, *MCS*, *IDI*, *MK*, and *PMK*, displayed syntenic relationships in most Asteraceae species (Figs S13, S15–S18). However, genes such as *HDR*, *HMGS*, and *HDS* exhibited synteny only among a limited number of species. Notably, *CMK* in *L. sativa* showed synteny exclusively with *A. annua*, while *MPD* and *CMS* were syntenic only with *S. marianum* and with *S. marianum* plus *C. morifolium*, respectively (Fig. S19). These limited syntenic relationships suggest poor conservation of these genes between Asteraceae and outgroup species, implying that the terpenoid biosynthetic pathway in Asteraceae may have undergone lineage-specific evolutionary changes. Despite variations in the conservation of upstream genes in the sesquiterpenoid synthesis pathway among *L. sativa*, *C. tinctorius*, and *S. marianum*, the three species share highly similar upstream gene sequences (Fig. 3C; Figs S13–S19; Table S11). The high degree of sequence similarity, combined with comparable expression profiles, supports the notion that these species may utilize conserved biosynthetic routes for terpenoid production, reflecting a common evolutionary origin.

### Identification and analysis of transcription factors regulating *CtTPS55* in *C. tinctorius*

**Figure 4:**
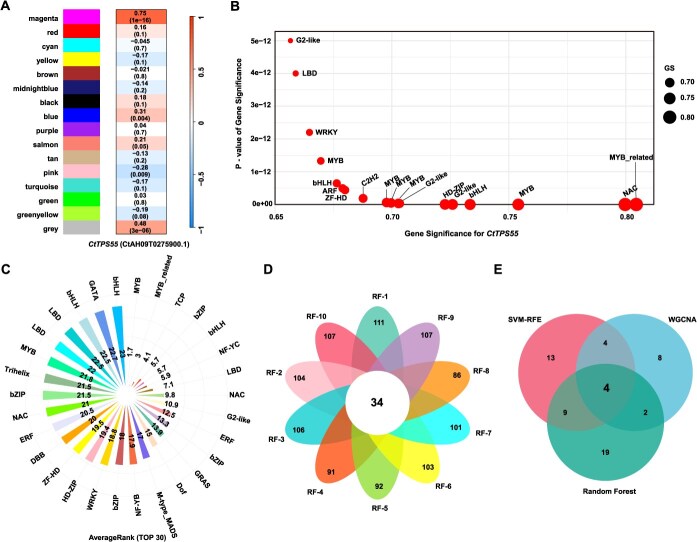
Construction of the co-expression gene network containing *CtTPS55* (CtAH09T0275900.1), and screening of TFs significantly associated with *CtTPS55*. (A) Identification of modules associated with *CtTPS55* using 87 transcriptome samples. (B) TFs that are co-expressed with *CtTPS55*. (C) The top 30 TFs associated with *CtTPS55* obtained using SVM-RFE. (D) TFs associated with *CtTPS55* were screened out by RF. (E) Venn diagram of the TFs identified by the three methods.

Based on the observed functional differences in the caryophyllene-type sesquiterpene BGCs between *C. tinctorius* and *S. marianum*, we further explored the potential transcriptional regulation of CPS gene expression in *C. tinctorius* by analyzing coexpressed TFs. We applied integrative computational approaches, including weighted gene coexpression network analysis (WGCNA), random forest (RF), and support vector machine recursive feature elimination (SVM-RFE), to identify TFs regulating the *C. tinctorius* CPS gene (CtAH09T0275900.1, *CtTPS55*). A total of 2079 TFs were identified in *C. tinctorius* through PlantTFDB. Based on transcriptome data from 87 transcriptome samples (SRP272164), WGCNA identified 16 coexpression modules. Correlation analysis revealed that the magenta module was highly positively correlated with *CtTPS55* (*r* = 0.75, *P* = 1 × 10^−16^), with 72 TFs coexpressed within this module (Fig. S20). Further correlation analysis between gene expression and phenotypic data identified 18 TFs significantly associated with *CtTPS55* (*GS* > 0.65; *P* < 1 × 10^−10^). Among them, CtAH12T0073900.1 showed the highest correlation (MYB_related, *GS* = 0.8045, *P-GS* = 6.27 × 10^−21^), followed by CtAH11T0105000.1 (NAC, *GS* = 0.7997, *P-GS* = 1.56 × 10^−20^) (Fig. 4B). In addition, using the same transcriptome data, RF and SVM-RFE were used to refine the selection of key TFs. Among the top 30 TFs identified by SVM-RFE, CtAH12T0169700.1 (MYB, Average rank = 1.7, *r* = 0.7541, *P* = 3.44 × 10^−17^) ranked the first (Fig. 4C, Table S12). Ten independent RF models, each initialized with a unique random seed, consistently achieved R^2^ > 0.9, indicating the model’s predictive reliability. A total of 34 TFs were identified through cross-iteration intersection analysis, highlighting their robustness as key regulators. (Fig. 4D, Table S13). By integrating results from WGCNA and the two machine learning methods (RF, SVM-RFE) through a Venn diagram, four key TFs were consistently identified by all methods which belonged to the LBD, MYB, NAC, and MYB_related families and were regarded as significantly associated with *CtTPS55* (Fig. 4E, Table S14). Based on the expression patterns of four key TFs and *CtTPS55* in different tissues of *C. tinctorius*, we found that CtAH12T0169700.1 and *CtTPS55* are expressed in roots, stems, leaves, and flowers, with notably high expression levels in roots (Fig. S21). This indicates that the MYB TF may play a regulatory role in the expression of *CtTPS55* in the roots of *C. tinctorius*, while other TFs may have pleiotropy and may also regulate the expression of other genes.

### Potential regulatory relationship of TPS genes in *C. tinctorius* under light stress

*Carthamus tinctorius* is rich in TPS genes, and the terpenoids synthesized by these genes may provide a competitive ecological advantage. *Cis*-acting element analysis of the 2000-bp region upstream of *CtTPS* genes revealed an abundance of light-responsive elements (Fig. S22), suggesting their potential involvement in the response to light stress. To decipher the regulatory relationship of TPS genes in *C. tinctorius* under environmental stress, a multilayered GRN was constructed.

**Figure 5:**
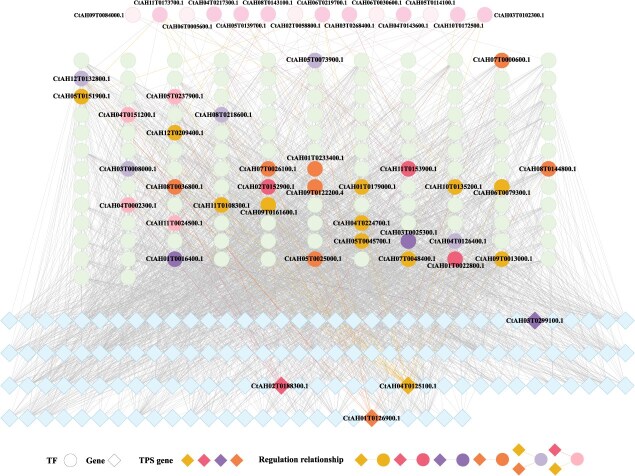
Gene regulatory network of *C. tinctorius* under different light intensities. Circles represent TFs, and diamonds represent genes. The *CtTPS* genes at the bottom layer are highlighted in different colors. TFs regulating the expression of *CtTPS* genes in the middle layer are also highlighted in different colors, with each color corresponding to a different regulatory relationship.

By performing transcriptome analysis under low, medium, and high light intensities through pairwise comparisons, 1338 (961 down/377 up), 2024 (908 down/1116 up), and 971 (251 down/720 up) differentially expressed genes (DEGs) were respectively identified (Fig. S23, Tables S15–S20). A total of 3195 DEGs were identified in the data of the three light intensities, including 8 *CtTPS* genes and 197 TFs (Fig. S24). GO enrichment analysis revealed that these DEGs were primarily associated with biological processes such as response to chitin, response to heat, response to water deprivation, response to water, and secondary metabolism (Fig. S25). In order to analyze the regulatory mechanism of TPS genes in response to light stress, we screened the pathways directly related to response to light intensity based on GO enrichment analysis, and integrated the biological processes in which TPS genes were significantly enriched, including response to wounding, hormone metabolic process, response to nutrient levels, and response to extracellular stimulus, as candidate pathways (FDR < 0. 05) (Table S21). After removing duplicates, 159 differentially expressed genes were retained for the construction of GRN. Based on these 159 genes and 197 TFs among the DEGs, a GRN was constructed, and 146 coexpressed genes, including four *CtTPSs*, were screened out through Pearson correlation analysis (CC > 0.8) (Table S22). Further partial correlation analysis (PCC < 0.3) showed that 134 TFs were involved in the regulation of these 131 coexpressed genes, of which 32 TFs regulated *CtTPSs* (Table S23). By analyzing the TF interaction network, it was determined that a significant coexpression relationship (CC > 0.8) existed among 130 TFs (Table S24). Using the same method, it was determined that 14 TFs at the top layer of the GRN regulated 130 TFs in the middle layer, among which nine were associated with the regulation of *CtTPS* genes (Table S25). Based on the GRN results, the hierarchical network consisted of 14 primary TFs regulating secondary TFs (134 TFs in the second layer), which in turn activated functional genes (131 genes in the third layer) to collectively respond to light stress. This network suggests a potential regulatory cascade through which light signals may be transmitted from global regulators to secondary regulators and ultimately to effector genes (Fig. 5).

To preliminarily verify the regulatory relationship of *CtTPS* in the multilayered GRN, we analyzed the expression profiles of *CtTPS* and TFs using another set of transcriptome data (samples of *C. tinctorius* under different light intensities and at different growth and development stages, SRP266264). The results showed that CtAH02T0188300.1 was relatively highly expressed in the first stage of low light intensity (LS) but had low expression levels under other light intensities; the TFs CtAH04T0151200.1 (WRKY) and CtAH02T0152900.1 (MYB), which shared the same expression pattern, might regulate this gene under low light conditions (Fig. S26A). The gene CtAH01T0126900.1 and the TF CtAH08T0218600.1 (B2) exhibited stable expression trends across various stages and light intensities (Fig. S26B), while the gene CtAH03T0299100.1 and the TF CtAH01T0016400.1 (WRKY) were relatively highly expressed in the first stage of high light intensity (HS) (Fig. S26C). In addition, there were differences in the expression between the gene CtAH04T0125100.1 and the screened TFs, indicating that these TFs might be involved in regulating the expression of this gene as well as other genes simultaneously (Fig. S26D).

## Discussion

Previous studies have demonstrated that the genomes of >40 Asteraceae species have been sequenced, facilitating functional genomics research. However, systematic evolutionary analysis of the TPS gene family across Asteraceae remains limited. In this study, the phylogenetic tree of 19 Asteraceae species was constructed, and the TPS gene family was analyzed. As a result, the differentiation time of Asteraceae species and the distribution characteristics of the TPS gene family were clarified. It was found that Asteraceae diverged from other families ~74.03 MYA and could be further classified into three subfamilies (Fig. 1A). Among the 19 Asteraceae species, TPS genes were mainly distributed within the TPS-a and TPS-b subfamilies, which may be associated with the rich diversity of terpenoid compounds in these species. The expansion of these gene families primarily occurred through tandem and segmental duplications (Fig. 1B, C, Fig. S4). These compounds not only account for the widespread aromaticity of Asteraceae species but also serve as important medicinal components [47]. TPS genes generated through tandem and segmental duplications are expressed in various tissues, contributing to terpene biosynthesis and likely promoting terpene accumulation.

Despite advances in plant BGC research, only a few have been functionally validated, and studies on BGCs in Asteraceae are particularly scarce. In this study, PlantiSMASH was used to systematically identify BGCs in 19 Asteraceae species. Caryophyllene-type sesquiterpenoid BGCs were identified in *C. tinctorius*, *S. marianum*, *T. mongolicum*, *T. kok-saghyz*, *A. annua*, *C. seticuspe*, *C. morifolium*, *E. breviscapus*, *B. alba*, and *D. pinnata* (Fig. S8). Additionally, a BGC responsible for the synthesis of germacrene A acid was discovered in *L. sativa*. This cluster consists of two GAS genes, one GAO gene, and one CoA-ligase, with CoA-ligase not being expressed in the roots, stems, and leaves of *L. sativa* (Fig. S12). Germacrene A acid, the metabolite produced by this BGC, serves as the biosynthetic precursor of lactucin and lactucopicrin, two sesquiterpene lactones that impart bitterness to lettuce and possess notable nutritional and medicinal properties [48]. This finding underscores the critical role of this BGC in germacrene A acid biosynthesis. Through synteny analysis and gene duplication pattern identification, it was revealed that the caryophyllene-type sesquiterpene BGCs in *C. tinctorius* and *S. marianum* likey originated from the tandem repeat aggregation of CPS genes and redox enzymes, while certain caryophyllene-type sesquiterpene BGCs in *C. morifolium* originated from WGD events (Fig. 2). Taken together, these results suggest that BGC evolution in Asteraceae is driven by gene tandem duplication and WGD events, with CPS genes being conserved in the common ancestor of Asteraceae [49]. Previous studies have shown that miltiradiene BGCs are highly conserved across seven Lamiaceae species, with minimal interspecies disruptions [32]. Similarly, collinear leonurine BGCs were identified in five Lamiaceae species, supporting the hypothesis that their common ancestor harbored primordial frameworks for both miltiradiene and leonurine BGCs, which were vertically inherited and preserved during species divergence [50]. In contrast, our findings suggest that the common ancestor of Asteraceae likely lacked preformed caryophyllene BGCs. Instead, these clusters appear to have emerged independently in different species through mechanisms such as tandem duplication and WGD. This evolutionary pattern may explain the observed low conservation of BGCs within Asteraceae.

Through synteny analysis of 11 Asteraceae species and two outgroup species, the evolutionary patterns of the terpenoid biosynthesis pathway were investigated. The results revealed varying degrees of gene loss among Asteraceae species and low conservation of related genes between outgroups and Asteraceae, suggesting that terpenoid synthesis genes in Asteraceae have undergone lineage-specific evolutionary processes. Although conservation of upstream sesquiterpenoid genes differed among *L. sativa*, *C. tinctorius*, and *S. marianum*, these species exhibited highly similar gene sequences and convergent expression patterns (Figs S13–S19, Table S11). Together, these findings indicate that Asteraceae species likely synthesize terpenoid compounds through conserved biosynthetic pathways sharing a common evolutionary origin.

Expression profile analysis of the CPS gene in BGCs revealed that *CtTPS55* in *C. tinctorius* was expressed in roots, stems, and leaves, with the highest expression observed in roots (Fig. 3). To explore the regulatory relationship of *CtTPS55* in *C. tinctorius*, WGCNA and two machine-learning methods were employed to identify key TFs regulating the *CtTPS55* gene. CtAH12T0169700.1 was identified as a potential regulator of CtTPS55 expression in roots (Fig. 4, Fig. S21). It was reported that seven TFs regulating the expression of TPS gene in *Dendrobium officinale* were screened by a similar screening method, which belonged to the bHLH, ERF, G2, GRAS, MYB, and NAC families [51]. These findings suggest that MYB may play a conserved role in plant adaptation to environmental stress, such as light stress.

In comparison with other Asteraceae species, *C. tinctorius* exhibits the highest relative abundance of TPS genes in its genome. We hypothesize that terpenoid metabolites regulated by TPS genes may mediate adaptive responses in *C. tinctorius*. The 73 TPS genes identified in this species harbor abundant light-responsive *cis*-elements in their promoter regions, indicating a potential role in light stress response (Fig. S22). By constructing a multilayered GRN, the regulatory relationship of *C. tinctorius* under different light intensities was analyzed. The results showed that a total of 3174 genes in *C. tinctorius* were affected by light stress, including 8 *CtTPS* genes and 197 TFs. In the regulatory network, nine primary TFs exert regulatory effects on 32 secondary TFs, which in turn activate four *CtTPS* genes, orchestrating a coordinated response to light stress (Fig. 5). These findings indicate that *CtTPS* genes may mediate plant responses to light stress by regulating terpene biosynthesis. This process integrates wound repair, phytohormone signaling crosstalk, nutrient allocation optimization, and extracellular chemical signaling (Table S21), thereby enhancing the ecological competitiveness of *C. tinctorius* under abiotic stress.

This study was based on high-quality genomic data from 19 Asteraceae species that met stringent inclusion criteria. However, the limited taxonomic scope may not fully capture the extensive evolutionary diversity within the family, which could constrain the generalizability of our conclusions. Future research should incorporate more species to enhance the robustness of the evolutionary framework and the biological relevance of the findings. In addition to the limitations in taxonomic coverage, this study also has methodological constraints. It primarily relies on genomic and transcriptomic datasets obtained from public databases and employs a range of bioinformatics tools and algorithms for gene identification, functional prediction, and evolutionary inference. While experimental validation of the bioinformatics predictions has not yet been conducted, the study remains valuable in several respects. The functional genes identified through sequence alignment and homology-based screening constitute a focused set of candidates for future experimental validation, thereby reducing the time and resource demands of gene discovery. Furthermore, the coexpression relationships inferred from transcriptomic data offer preliminary insights into biological processes such as stress responses and terpenoid metabolism, providing a basis for future experimental studies on regulatory mechanisms. In future work, we will perform functional experiments to verify the identities and roles of the predicted genes and their regulatory interactions, thereby deepening our understanding of the underlying biological mechanisms.

## Conclusions

This study provides insights into the evolutionary trajectories of TPS genes and BGCs in Asteraceae. Our findings demonstrate that gene tandem duplication and WGD events have played pivotal roles in the expansion and diversification of sesquiterpenoid BGCs, particularly those encoding caryophyllene-type compounds. The identification of conserved upstream terpenoid biosynthesis genes across Asteraceae species underscores a shared evolutionary history in terpene metabolism. Furthermore, the discovery of light-responsive TPS gene regulatory networks highlights the adaptive significance of terpenoid biosynthesis in mediating plant–environment interactions. These findings not only advance our understanding of BGC evolution in Asteraceae but also lay a foundation for metabolic engineering strategies to enhance the production of bioactive sesquiterpenoids.

## Methods

### Phylogenetic reconstruction and divergence time estimation

Low-copy orthologous gene families across 25 species (Table S26) were identified using OthroFinder (v 2.5.4) [40]. To ensure data reliability, low-copy gene families were selected based on the criteria that each species contained no more than three paralogous genes and that at least 90% of species within each gene family had more than one copy [52]. Subsequently, a phylogenetic tree was constructed using IQ-TREE (v 2.1.4) [53] based on the identified low-copy gene families. To estimate the divergence time of Asteraceae species, the evolutionary time of species was estimated by using MCMCtree in PAML (v 4.10.7) [43, 54]. The sampling parameters were set as follows: burn-in = 400 000, sampfreq = 10, and nsample = 100 000, ensuring robust time estimation [52]. Moreover, we calibrated the evolutionary time data for Asteraceae species by referring to the TimeTree website (https://timetree.org/home) [44], using 15 fossil constraints to calibrate key nodes in Asteraceae (Table S27). To further explore the evolutionary dynamics of Asteraceae species, gene family expansion and contraction were analyzed using CAFÉ (v5.1.0), providing insights into the functional diversification of gene families over evolutionary time [55].

### Phylogenetic analyses of TPS gene families

To identify TPS family members, HMM profiles (PF01397, PF03936) were downloaded from the InterPro database (https://www.ebi.ac.uk/interpro/entry/pfam/#table) [56]. Protein sequences from 25 species were retrieved using HMMER tool (v 2.3.2) with default parameters [57]. Candidate TPS sequences were filtered using a threshold of *E-value* < 1 × 10^−5^. For comparative analysis, *Arabidopsis thaliana* TPS gene protein sequences were retrieved from the TAIR database (https://www.arabidopsis.org/). Sequence alignment between *A. thaliana* TPS protein sequences and those of the 25 species was conducted using MAFFT (v 7.505) [58]. A phylogenetic tree was constructed using IQ-TREE (v 2.1.4) [53]. The maximum likelihood method was employed to infer phylogenetic relationships based on the sequence alignments. Finally, the phylogenetic tree was visualized using the iTOL (https://itol.embl.de/) online tool.

### Prediction, phylogenetic analysis, and synteny analysis of terpene BGCs in Asteraceae species

Potential BGCs for 19 Asteraceae species were predicted by using a local version of the PlantiSMASH in conjunction with the genome files (FASTA format) and annotation files (GFF format) of these Asteraceae species [59]. PlantiSMASH was utilized to identify and annotate gene clusters associated with terpenoid biosynthesis [34]. TPS genes involved in terpene synthesis were extracted from all the predicted terpene BGCs, with a particular emphasis on the clusters related to sesquiterpene synthesis. Protein sequences of TPS genes with known functions in *L. sativa*, *C. cardunculus*, and *H. annuus* were downloaded from the NCBI database to ensure the coverage of sesquiterpene synthesis-related genes with known functions. The protein sequences of TPS genes were aligned and then underwent phylogenetic analysis using the IQ-TREE (v 2.1.4) tool to construct an evolutionary tree. Genes clustered in the same group were assumed to have similar functions, allowing for a preliminary function classification. Based on the results of the evolutionary tree, the TPS genes were functionally categorized, with special attention paid to those related to sesquiterpene synthesis. Synteny analysis was carried out by using the MCScan (Python) from the JCVI library [45]. This analysis revealed insights into the conservation, rearrangement, and expansion of gene clusters among species.

### Transcriptome analysis and TPS gene expression profiling

Transcriptome data from root, stem, and leaf of *V. vinifera*, *P. grandiflorus*, *C. tinctorius*, *L. sativa*, and *S. marianum* were collected from NCBI [60, 61]. Quality control of the transcriptomic data was carried out using Trim Galore (v 0.6.10) [62] to guarantee the accuracy and reliability of the data (parameters: –phred33 -q 30 –stringency 1 –length 50). FastQC (with default parameters) was used to assess the quality of transcriptomic data, ensuring that the data quality met the requirements of the study. Sequence alignment was performed using Hisat2 (v 2.2.1) [63], and data format conversion was conducted using Samtools (v 1.21) [64] for subsequent processing. Gene expression levels were calculated using FeatureCounts (v 2.0.6) tool [65]. The raw counts were imported into DESeq2 for processing, and expression levels were normalized using fragments per kilobase of exon model per million mapped fragments (FPKM) values [66]. Finally, gene expression patterns across different tissue types were visualized using the R package ggplot2. A comparative analysis of TPS gene expression across the tissues was also conducted.

### Identification of feature transcription factors for sesquiterpene accumulation

To identify feature TFs regulating the expression of *C. tinctorius* terpene synthase genes, a combination of three methods, namely WGCNA [67], RF, and SVM-RFE, was applied for key TF screening [51]. First, 87 RNA-seq datasets of *C. tinctorius* (SRP272164) were downloaded from the SRA database of NCBI and normalized. Through the PlantTFDB website (https://planttfdb.gao-lab.org/prediction_result.php), 2079 TFs in *C. tinctorius* were identified. Subsequently, genes with a total expression of zero were excluded, leaving 1679 TFs for subsequent analysis with TPS genes. The expression data of the gene *CtTPS55* was used as the phenotype, and the expression data of 1679 TFs in *C. tinctorius* served as the predictor variables.

Next, the WGCNA toolkit in R was utilized to conduct WGCNA. A minimum module size of 30 was selected to construct the coexpression module. The optimal soft-thresholding parameter was determined by calculating the scale-free topology-fitting index and average connectivity to construct a scale-free network. Based on the gene correlation matrix, the topological overlap matrix was constructed to quantify the network connectivity between genes. TFs with a significant correlation with *CtTPS* genes were screened based on the criteria of a *P-value* <1e-5 and a Gene Significance (GS) ≥0.65 [51, 67]. Finally, the results of the WGCNA analysis were network-visualized using Cytoscape software to display the coexpression relationship between genes.

The RF algorithm combined with Boruta was used to screen key features and construct a regression model. Eighty-seven transcriptomic samples were split into a 75% training set and 25% test set, with random assignment repeated 10 times using different random seeds to ensure result stability. The Boruta algorithm employed 300 iterations and a strict significance threshold of 0.01 (with Bonferroni multiple-test correction) to identify Confirmed and Tentative features. The RF parameter mtry was optimized via 10-fold cross-validation repeated five times, *t* aiming to minimize mean squared error (MSE). Model performance was evaluated using *R*^2^, and feature importance was ranked to derive critical feature subsets for gene expression analysis. The final list of TFs identified by the RF method was derived from the intersection of results across 10 independent runs.

SVM-RFE was applied to screen key features associated with TPS gene expression levels. Using the kernlab and e1071 packages, a support vector regression (SVR) model with a radial basis function kernel was constructed. Penalty parameter (C = 0.1, 1) and kernel parameter (gamma = 0.1, 1) were optimized via 10-fold cross-validation. Recursive feature elimination was performed on a standardized feature matrix, with subset sizes increasing from 50 to the full feature set in steps of 100. Model performance was evaluated using root mean squared error (RMSE). The feature subset with the lowest RMSE was selected, and the most stable feature combinations were identified by averaging rankings of feature importance across cross-validation folds. Eventually, the top 30 TFs selected by SVM-RFE were considered key regulators.

Finally, the TFs obtained from the three methods were integrated, and the intersection was selected to reduce false-positive results and enhance the reliability and accuracy of the screening.

### Identification of genes related to sesquiterpenes biosynthesis

The protein sequences of *A. thaliana* involved in sesquiterpene biosynthesis were retrieved from the KEGG database. Using the BLASTp tool (*E-value* < 1e-5, identity >50%, score >200), homologous genes related to sesquiterpene biosynthesis were identified in *V. vinifera*, *P. grandiflorus, C. tinctorius*, *L. sativa*, and *S. marianum*. Subsequently, the expression levels of these genes in root, stem, and leaf tissues were visualized using the ggplot2 package, and a heatmap was generated. Finally, the sesquiterpene biosynthesis pathway diagram was drawn, providing a clear representation of the expression patterns of these genes in different tissues and their roles in the biosynthetic pathway.

### Construction of a multilayered hierarchical gene regulatory network using a partial correlation coefficient-based algorithm

The Bottom-up GGM is a multi-layered GRN construction strategy based on biological processes or pathways [68]. First, differential gene expression analysis was performed on the gene expression data from different sample groups under light stress conditions using DESeq2 to identify differentially expressed genes (*P* < 0.05, |log₂FoldChange| > 1). Subsequently, with *A. thaliana* as the reference genome, GO annotation was carried out for the *C. tinctorius* genes to identify GO categories associated with light stress. During the network construction process, based on the Bottom-up GGM algorithm, the identified pathway genes were set as the bottom layer of the network, while regulatory genes were placed in the upper layers. Next, the Pearson correlation coefficient between the bottom-layer genes was calculated to evaluate the correlation of gene expression. When the partial correlation coefficient (PCC) of a gene pair was ≥0.8, it was inferred that these genes might be regulated by the same TF. Subsequently, upper layer TFs are introduced, and further analysis is performed based on PCC [69]. The formula for calculating partial correlation coefficients is ${r}_{xy\mid z}=\frac{r_{xy}-{r}_{xz}{r}_{yz}}{\sqrt{\left(1-{r}_{xz}^2\right)\left(1-{r}_{yz}^2\right)}}$, where ${r}_{xy}$ is the correlation coefficient between genes x and y, and ${r}_{xz}$ and ${r}_{yz}$ are the correlation coefficients between the TF z and genes x and y, respectively. If the resulting PCC value was <0.3, it was considered that the regulation of this gene pair might be influenced by the TF. Through this strategy, TFs that directly regulate the bottom layer genes could be identified. These TFs were then treated as new bottom layer genes, and the above steps were repeated to construct higher level regulatory networks. Finally, the GRN with multilayer regulatory structures was visualized using Cytoscape software, which revealed the regulatory relationships and hierarchical structure between different genes.

## Supplementary Material

Web_Material_uhaf229

## Data Availability

The genome sequence and RNA-seq data analyzed in this study are publicly available, and the corresponding links are provided in Table S26.
