# Supplementary material for: Evolution of terpene synthases in the sesquiterpene biosynthesis pathway and analysis of their transcriptional regulatory network in Asteraceae
Source: Hortic Res. 2025 Sep 2;12(12):uhaf229. doi: 10.1093/hr/uhaf229 (PMC12682069; doi:10.1093/hr/uhaf229)
Supplement: Web_Material_uhaf229 [file web_material_uhaf229.zip › Supplementary Figures.pdf]

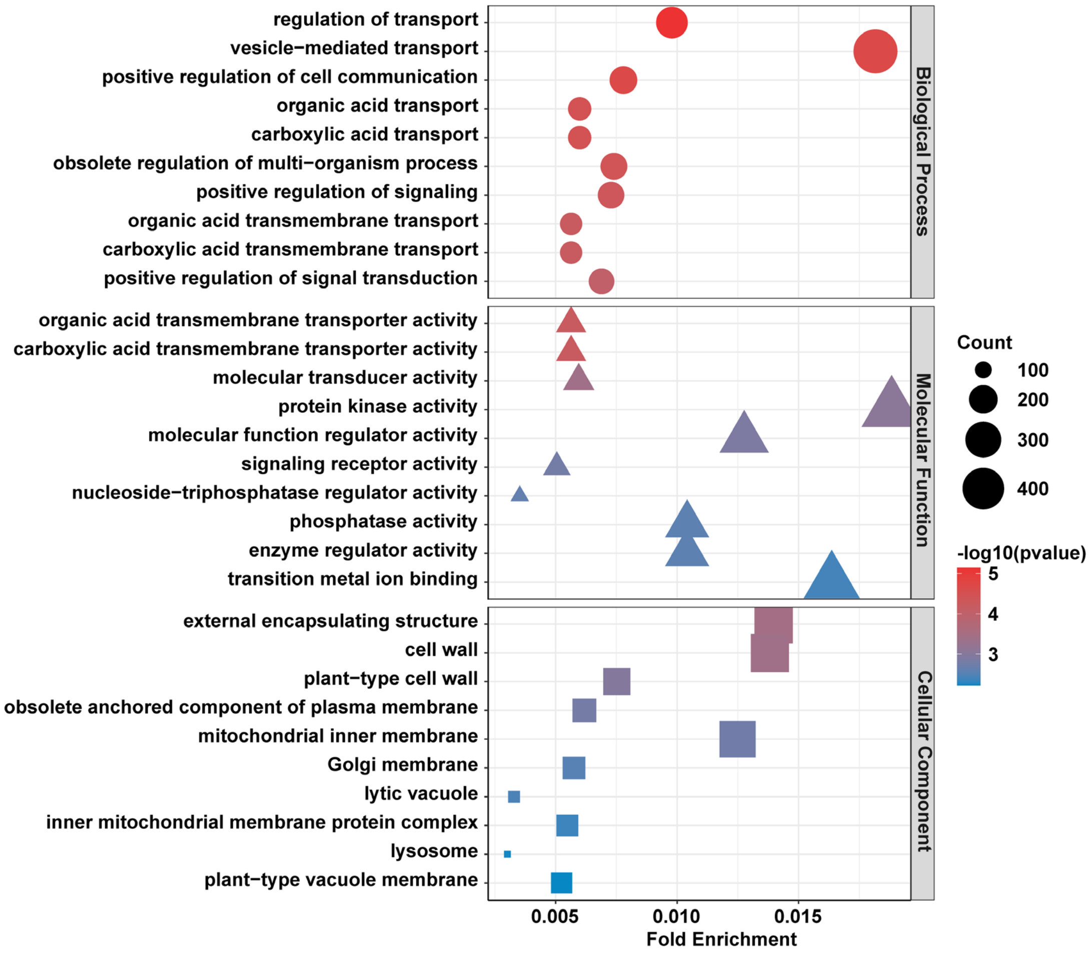


**Supplementary Figure 1**. GO enrichment analysis of expanded gene families in *C. morifolium*.

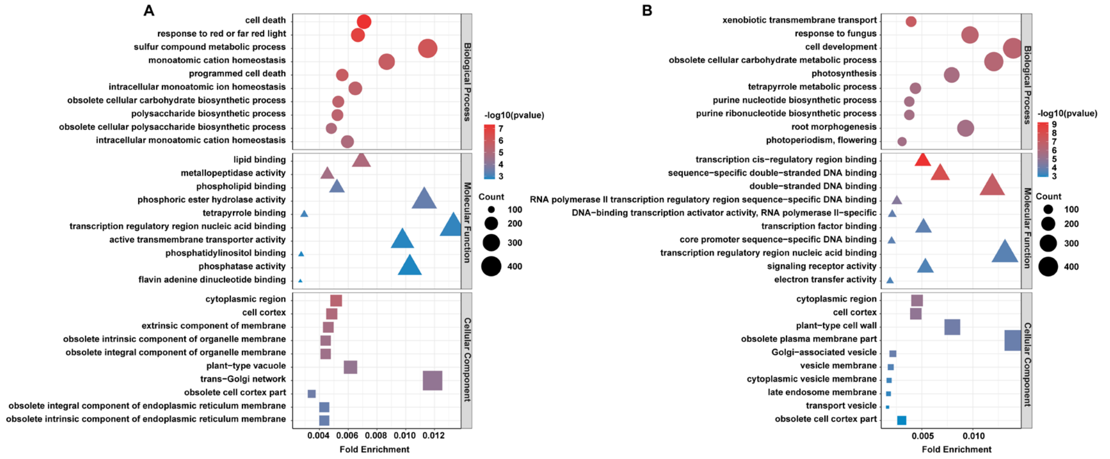


**Supplementary Figure 2**. GO enrichment analysis of *D. pinnata* and *B. alba* expanded gene families. (A) GO enrichment analysis of *D. pinnata* amplified gene families. (B) GO enrichment analysis of *B. alba* amplified gene families.

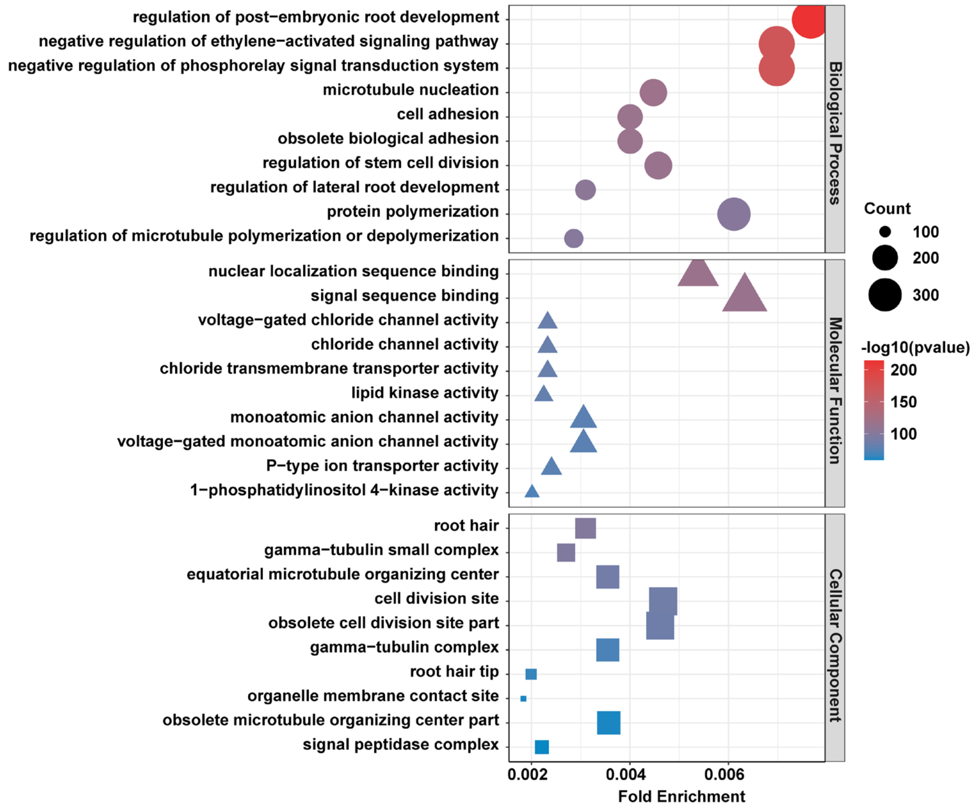


**Supplementary Figure 3**. Showing the results of GO enrichment analysis of 1,008 gene families expanded at the divergence nodes of Asteraceae and other outgroup species, including the enrichment entries and corresponding enrichment significance levels for three aspects: biological processes, molecular functions and cellular components.

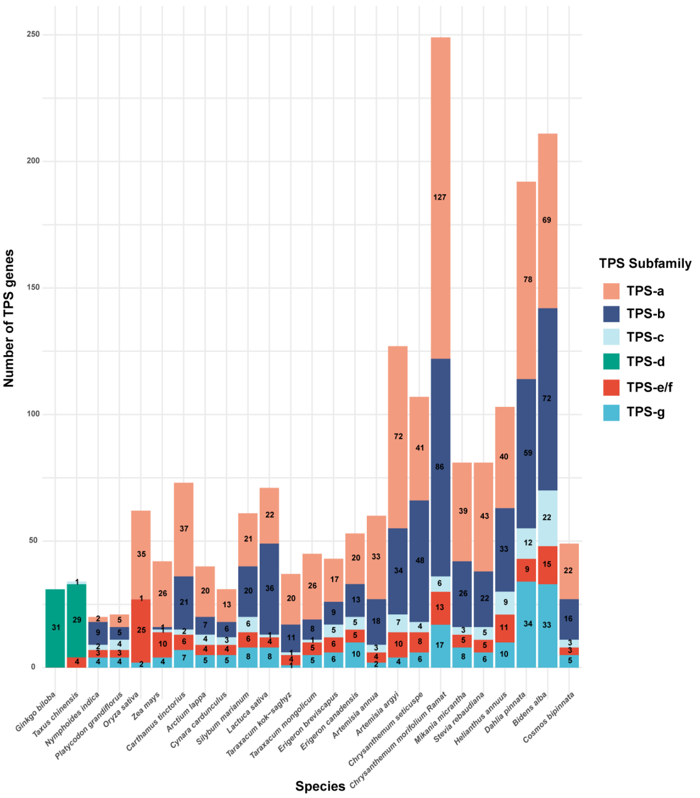


**Supplementary Figure 4**. Quantitative distribution of the different TPS gene subfamilies in 19 Asteraceae species and 6 outgroup species.

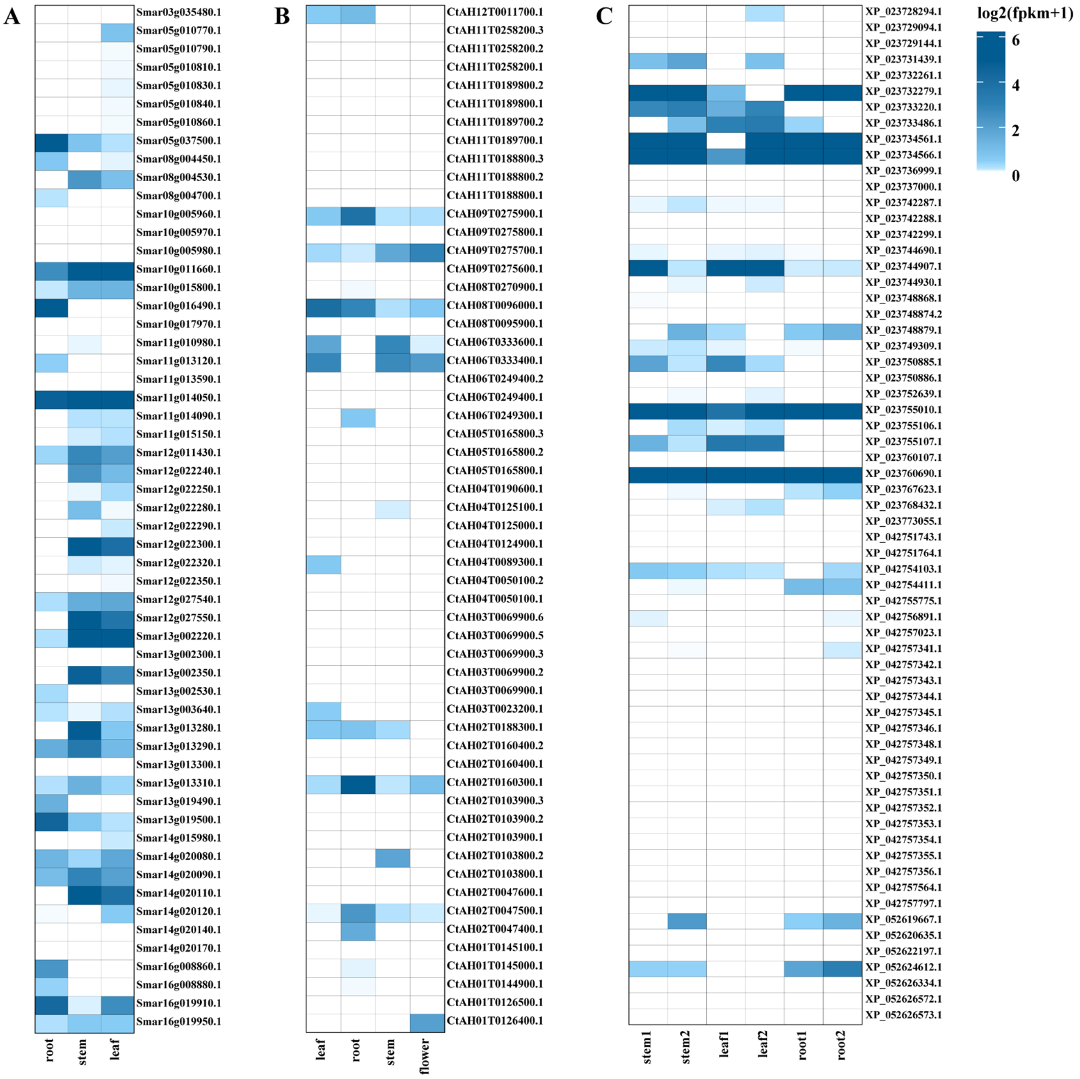


**Supplementary Figure 5**. Expression profiles of TPS genes formed by tandem and segmental duplications in different tissues of Asteraceae species. Panels A–C represent *S. marianum* (A), *C. tinctorius* (B), and *L. sativa* (C), respectively. Gene expression levels are shown as $\log_2$(FPKM + 1), reflecting the abundance of TPS transcripts in roots, stems, leaves, and flowers. RNA-seq data were obtained from the following NCBI BioProjects: PRJNA1021369 (A), PRJNA909037 (B), and PRJNA1103148 (C).

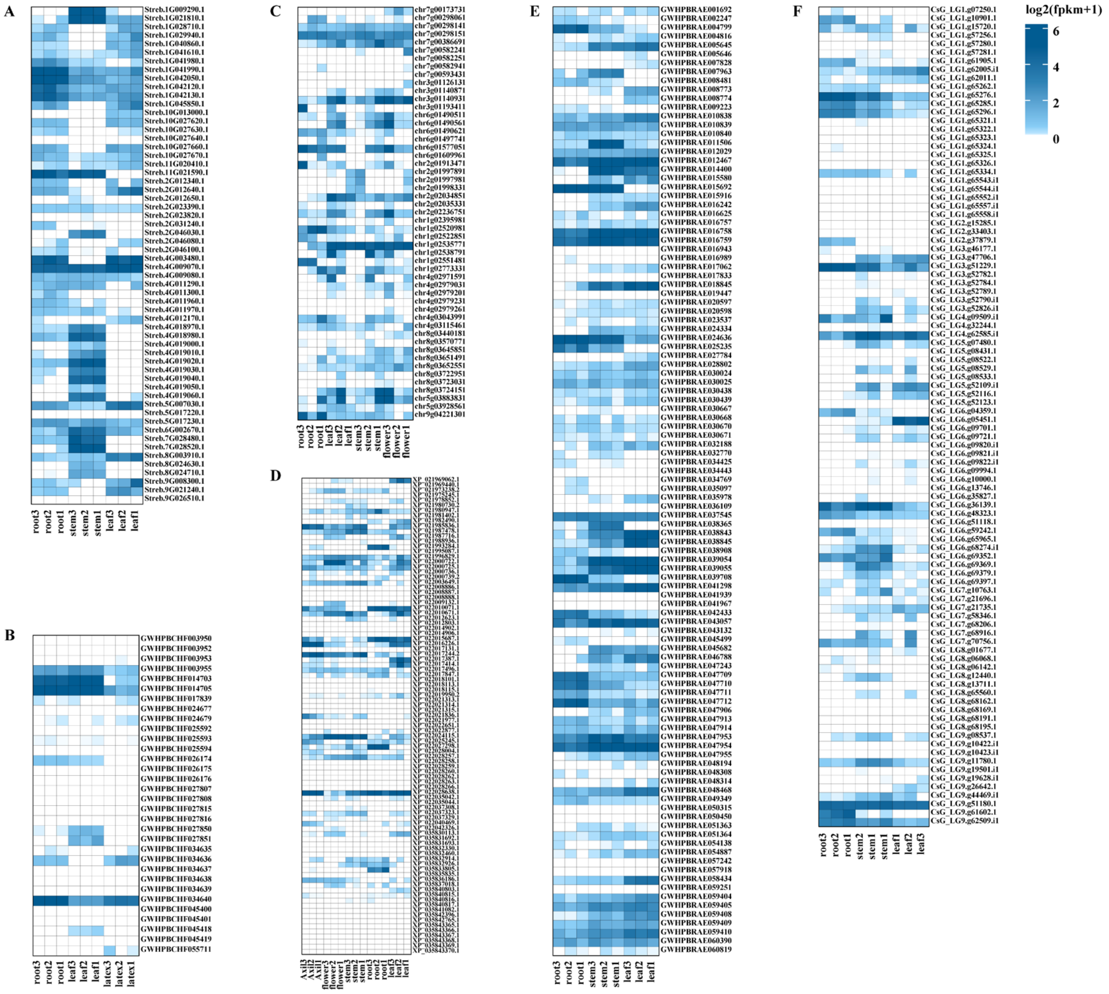


**Supplementary Figure 6**. Expression profiles of TPS genes derived from tandem and segmental duplication in different tissues of six Asteraceae species. Panels A–F correspond to *S. rebaudiana* (A), *T. kok-saghyz* (B), *A. annua* (C), *H. annuus* (D), *A. argyi* (E), and *C. seticuspe* (F), respectively. Gene expression levels are shown as $\log_2$(FPKM + 1). RNA-seq data were obtained from the following NCBI BioProjects: PRJNA705537 (A), PRJNA931633 (B), PRJNA752933 (C), PRJNA912670 (D), PRJNA722539 (E), and PRJNA1167174 (F).

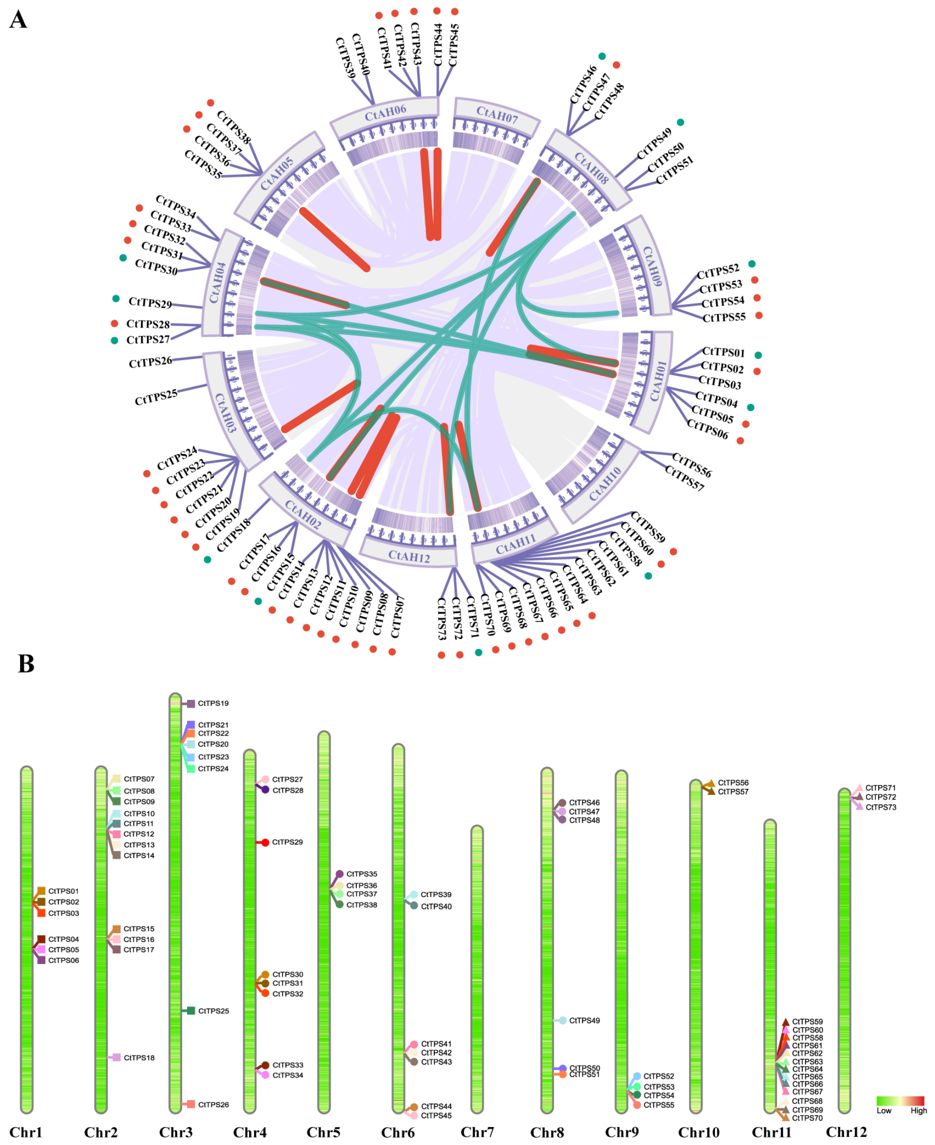


**Supplementary Figure 7**. Intraspecific collinearity relationships in *C. tinctorius* and the chromosomal localization of TPS genes. (A) A circos plot of the collinear relationships within *C. tinctorius*. TPS genes formed by segmental duplication are linked by green lines. Genes formed by tandem duplication are connected by red lines. (B) Visualization of the chromosomal locations of 73 TPS genes in *C. tinctorius*.

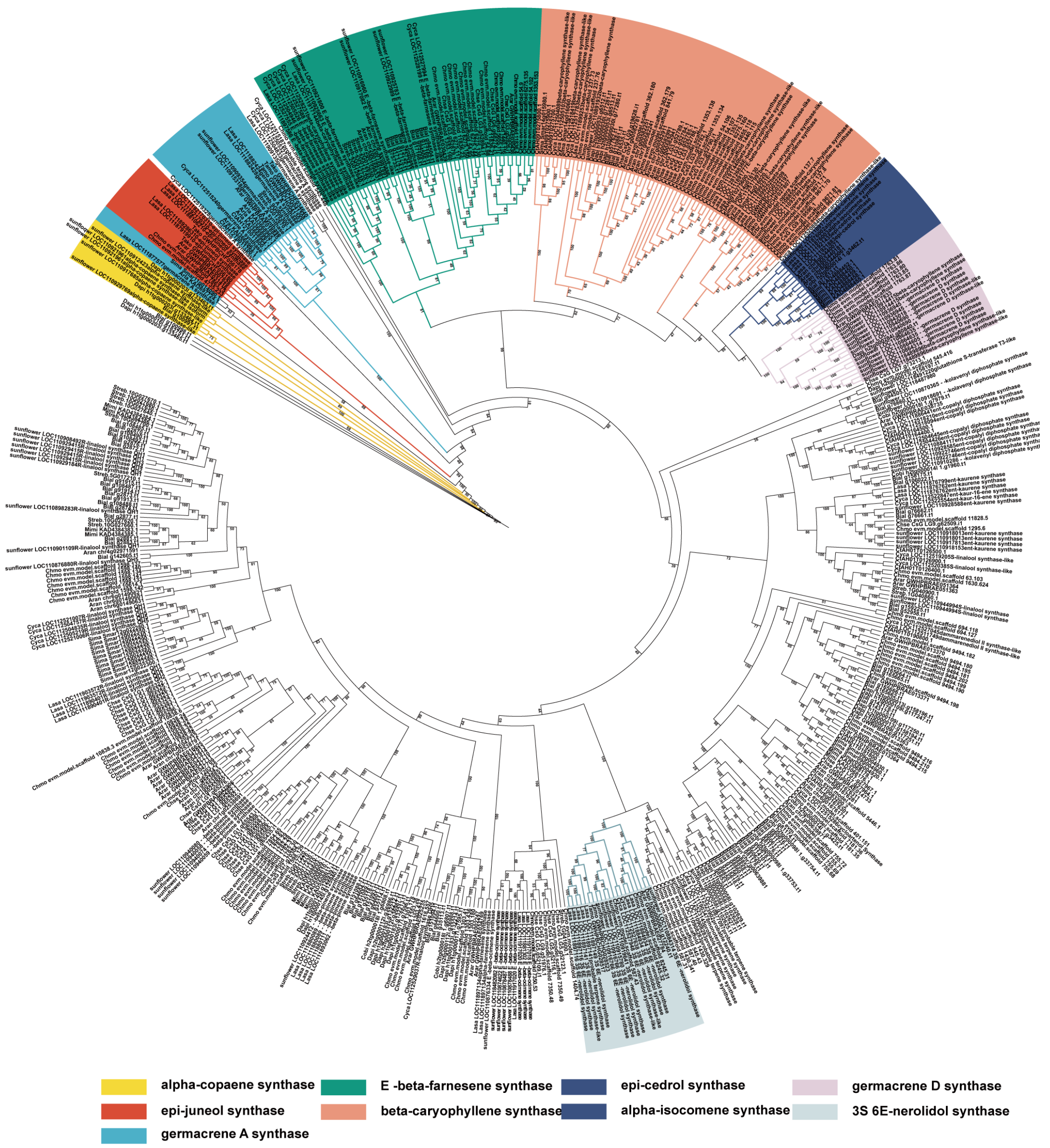


**Supplementary Figure 8**. Identification of genes with similar functions based on phylogenetic analysis. The phylogenetic tree was constructed using 558 TPS genes from BGCs of Asteraceae species. The highlighted branches represent the sesquiterpene synthase gene clade, whereas the remaining branches correspond to monoterpene, diterpene, and other terpene synthase genes.

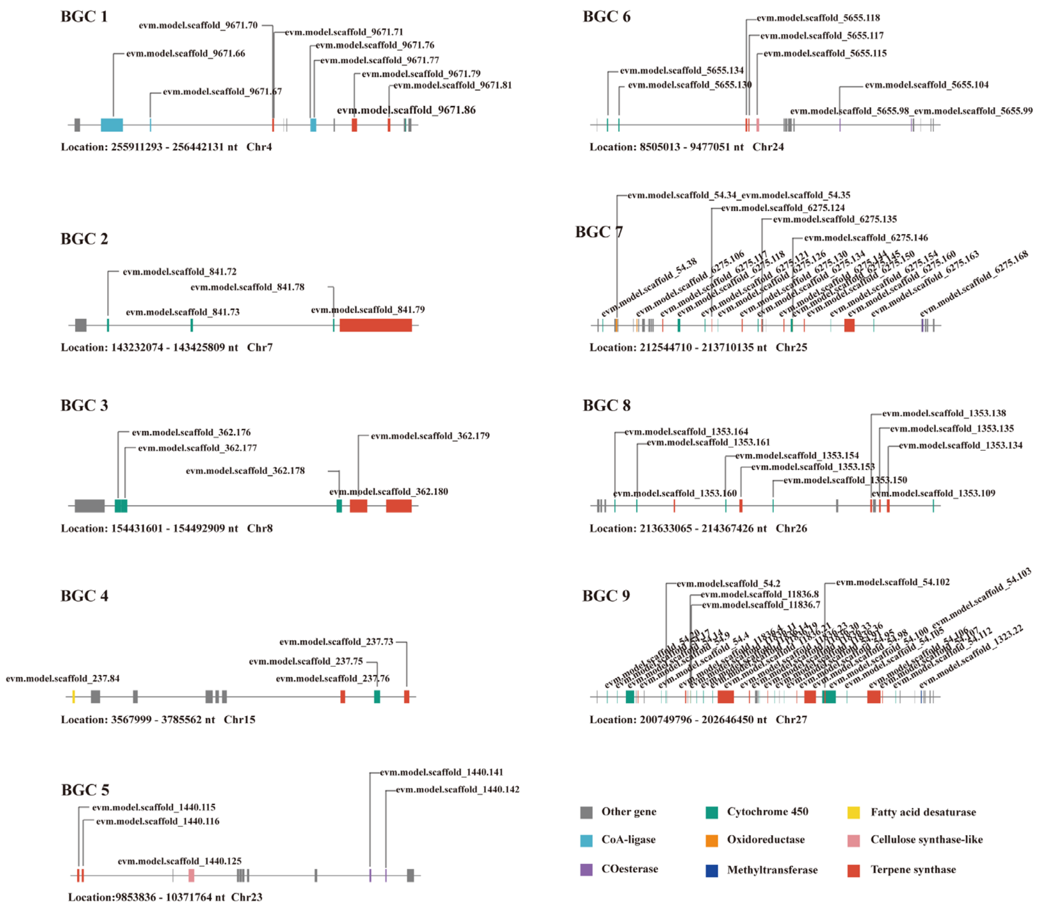


**Supplementary Figure 9**. Sesquiterpene biosynthetic gene clusters in *Chrysanthemum morifolium* Ramat.

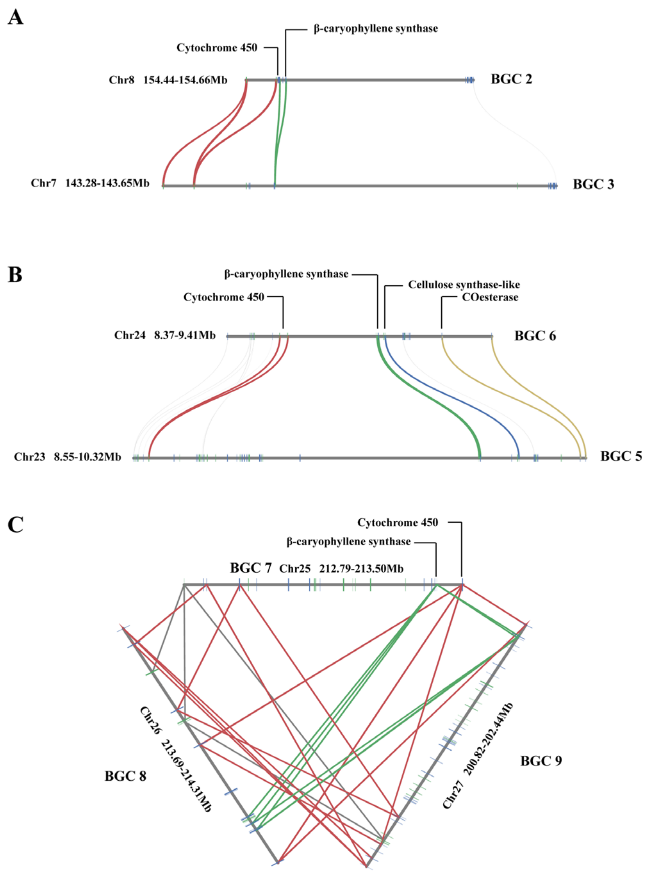


**Supplementary Figure 10**. Synteny relationships among different gene clusters in *Chrysanthemum morifolium* Ramat. (A) Synteny relationship between BGC2 and BGC3. (B**)** Synteny relationship between BGC5 and BGC6. (C) Synteny relationships among BGC25, BGC26, and BGC27.

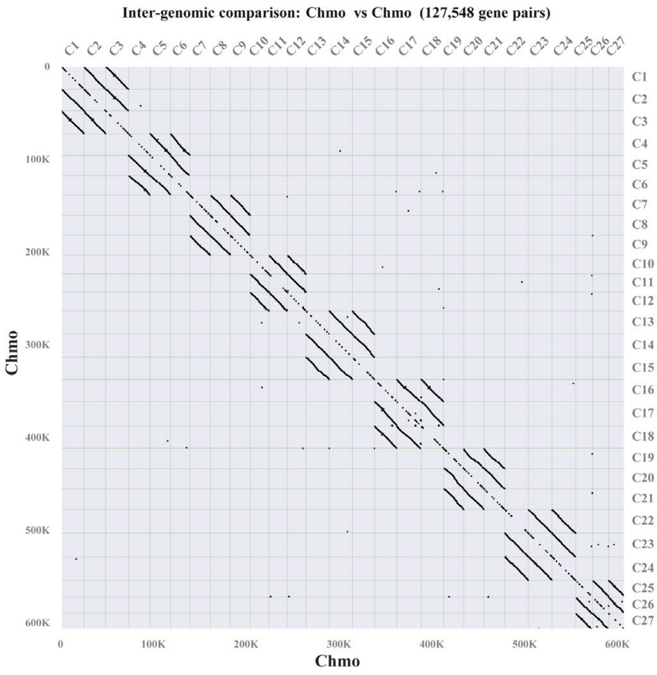


**Supplementary Figure 11**. Dot plot of covariate relationships within species of *Chrysanthemum morifolium* Ramat. Chmo: *Chrysanthemum morifolium* Ramat.

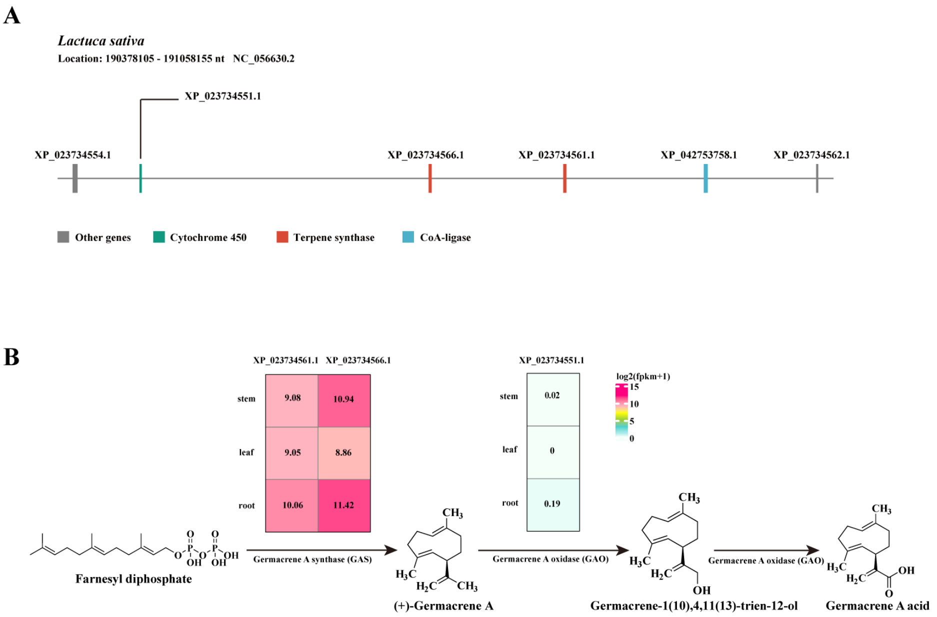


**Supplementary Figure 12**. Gene clusters in *Lactuca sativa* and the sesquiterpenoid biosynthesis pathway. (A) Gene clusters in Lactuca sativa. (B) The identification and expression levels of genes related to the biosynthetic pathway for sesquiterpenoid (Germacrene A acid) synthesis in Lactuca sativa were conducted. The expression levels (log10 (FPKM + 1)) of these genes were calculated, and the average values of two biological replicates were presented in the heatmap (SRP503378, NGS).

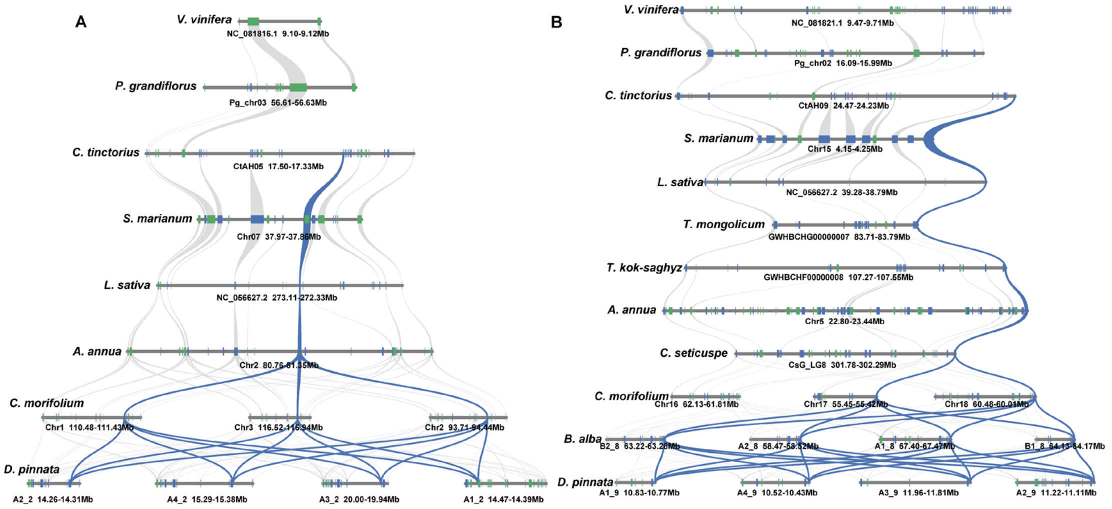


**Supplementary Figure 13**. Synteny relationships of the *ATOT* and *DXR* genes. (A) Synteny relationships of the *ATOT* gene among *V. vinifera, P. grandiflorus, L. sativa*, *C. tinctorius*, *S. marianum*, *A. annua*, *C. morifolium*, and *D. pinnata*. Rectangles represent annotated genes, with genes on the reverse strand shown in green and those on the same strand in blue. The lines connecting the syntenic *ATOT* genes are highlighted in blue. The grey lines represent the gene collinearity among the candidate species. (B) Synteny relationships of the *DXR* gene among *V. vinifera, P. grandiflorus, L. sativa*, *C. tinctorius*, *S. marianum*, *T. mongolicum*, *T. kok-saghyz*, *A. annua*, *C. seticuspe*, *C. morifolium*, *B. alba*, and *D. pinnata*. The lines connecting the syntenic *DXR* genes are highlighted in blue.

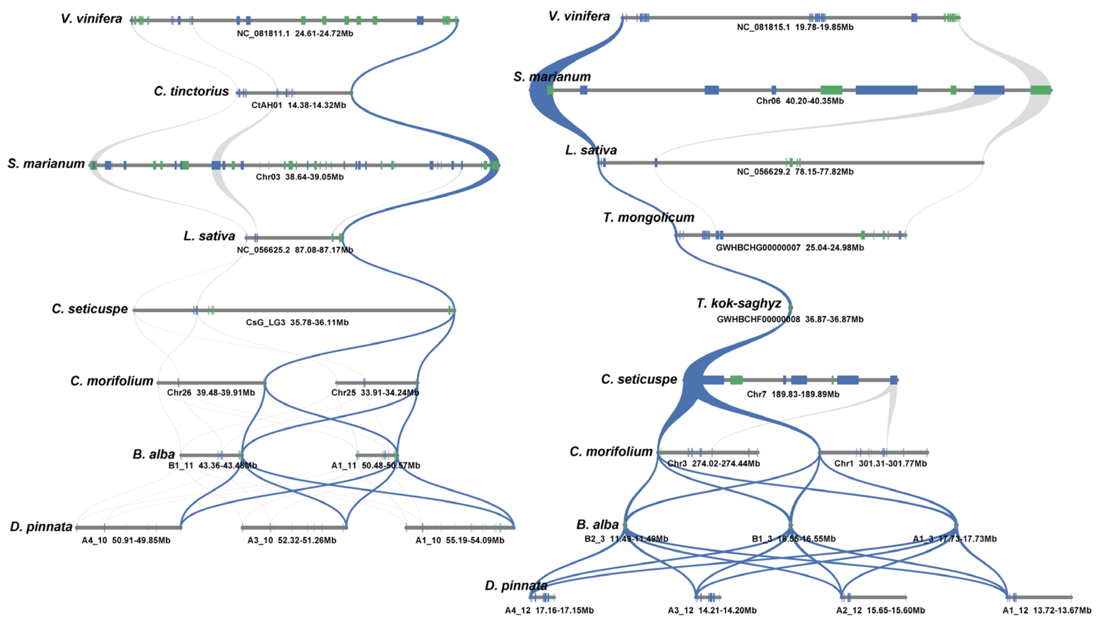


**Supplementary Figure 14**. Synteny relationships of the *DXS* gene. Rectangles represent annotated genes, with genes on the reverse strand indicated in green and those on the same strand in blue. The lines connecting the syntenic *DXS* genes are highlighted in blue. The grey lines represent the gene collinearity among the 13 species.

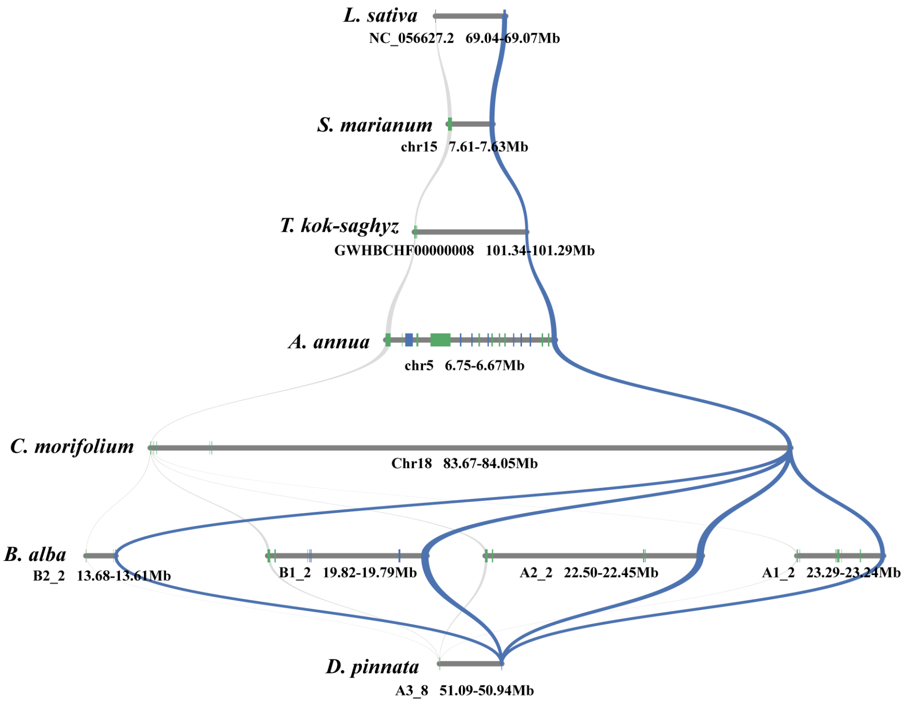


**Supplementary Figure 15**. Synteny relationships of the *FPPS* gene among seven plants of the Asteraceae family. Rectangles represent annotated genes, with genes on the reverse strand indicated in green and those on the same strand in blue. The lines connecting the syntenic *FPPS* genes are highlighted in blue. The grey lines represent the gene collinearity among the candidate species.

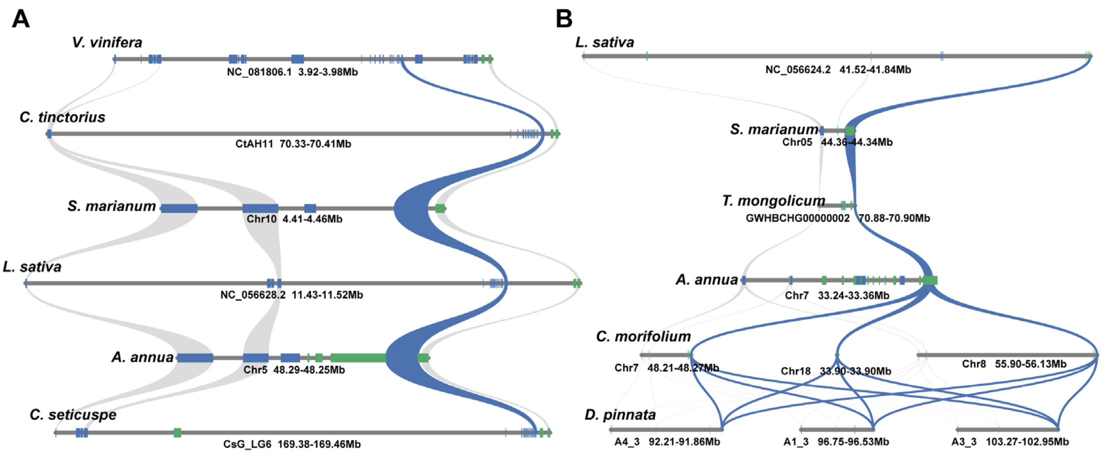


**Supplementary Figure 16**. Synteny relationships of the *HMGS* and *IDI* genes. (A) Synteny relationships of the *HMGS* gene among five Asteraceae species (*V. vinifera*, *L. sativa, S. marianum, T. mongolicum, A. annua, C. seticuspe*). The lines connecting the syntenic *HMGS* genes are highlighted in blue. (B) Synteny relationships of the *HMGS* gene among six Asteraceae species (*L. sativa, S. marianum, T. mongolicum, A. annua, C. morifolium, D. pinnata*). The lines connecting the syntenic *IDI* genes are highlighted in blue. Rectangles represent annotated genes, with genes on the reverse strand indicated in green and those on the same strand in blue. The lines connecting the syntenic genes are highlighted in blue. The grey lines represent the gene collinearity among the candidate species.

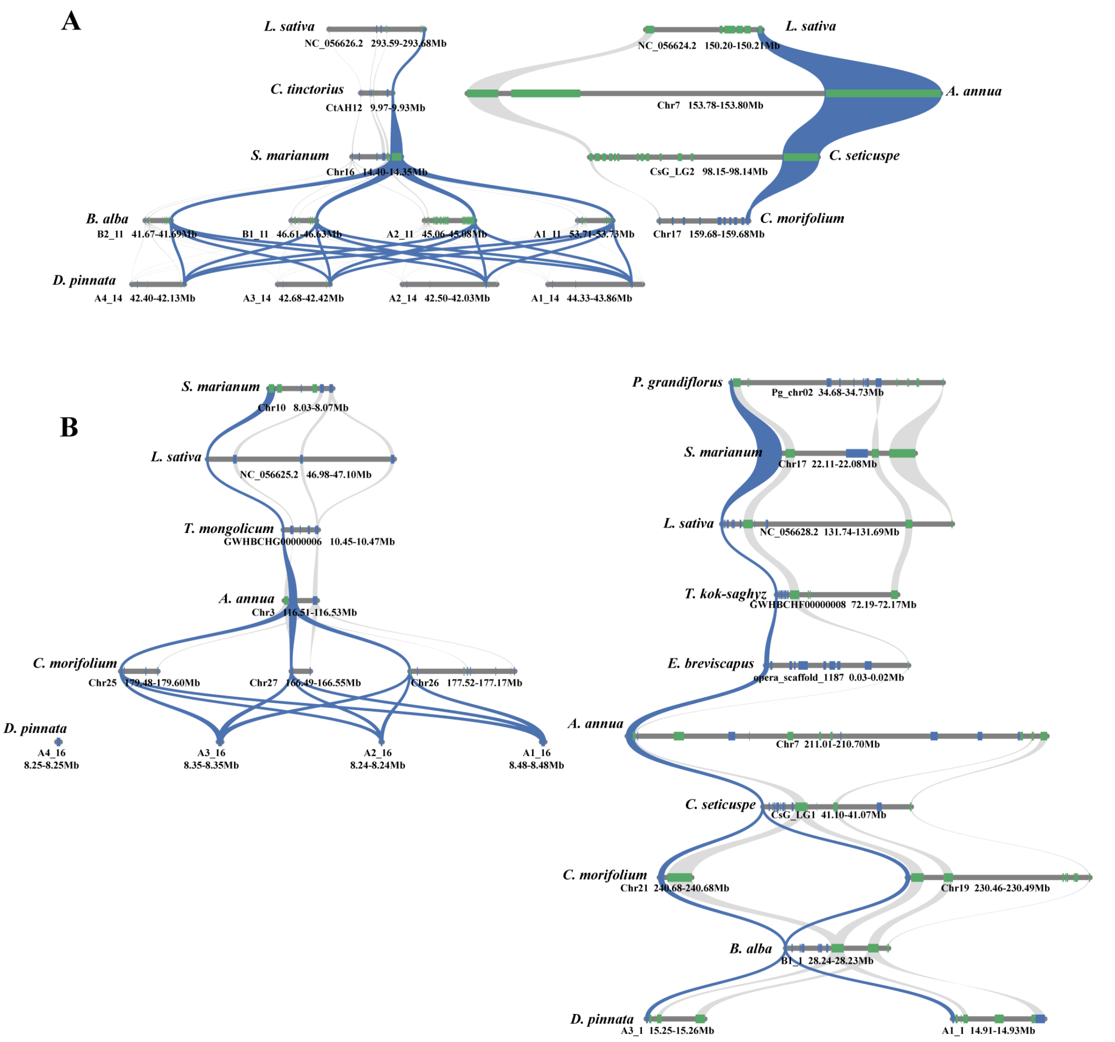


**Supplementary Figure 17**. Synteny relationships of the *MK* and *PMK* genes. (A) Synteny relationships of the *MK* gene among eight Asteraceae species (*L. sativa, C. tinctorius, S. marianum, B. alba, D. pinnata, A. annua, C. seticuspe, C. morifolium*). The lines connecting the syntenic *MK* genes are highlighted in blue. (B) Synteny relationships of the *PMK* gene among 11 Asteraceae species (*P. grandiflorus*, *L. sativa, S. marianum, T. mongolicum, A. annua, C. morifolium, D. pinnata, B. alba, C. seticuspe, E. breviscapus, T. kok-saghyz*). The lines connecting the syntenic *PMK* genes are highlighted in blue. Rectangles represent annotated genes, with genes on the reverse strand indicated in green and those on the same strand in blue. The lines connecting the syntenic genes are highlighted in blue. The grey lines represent the gene collinearity among the candidate species.

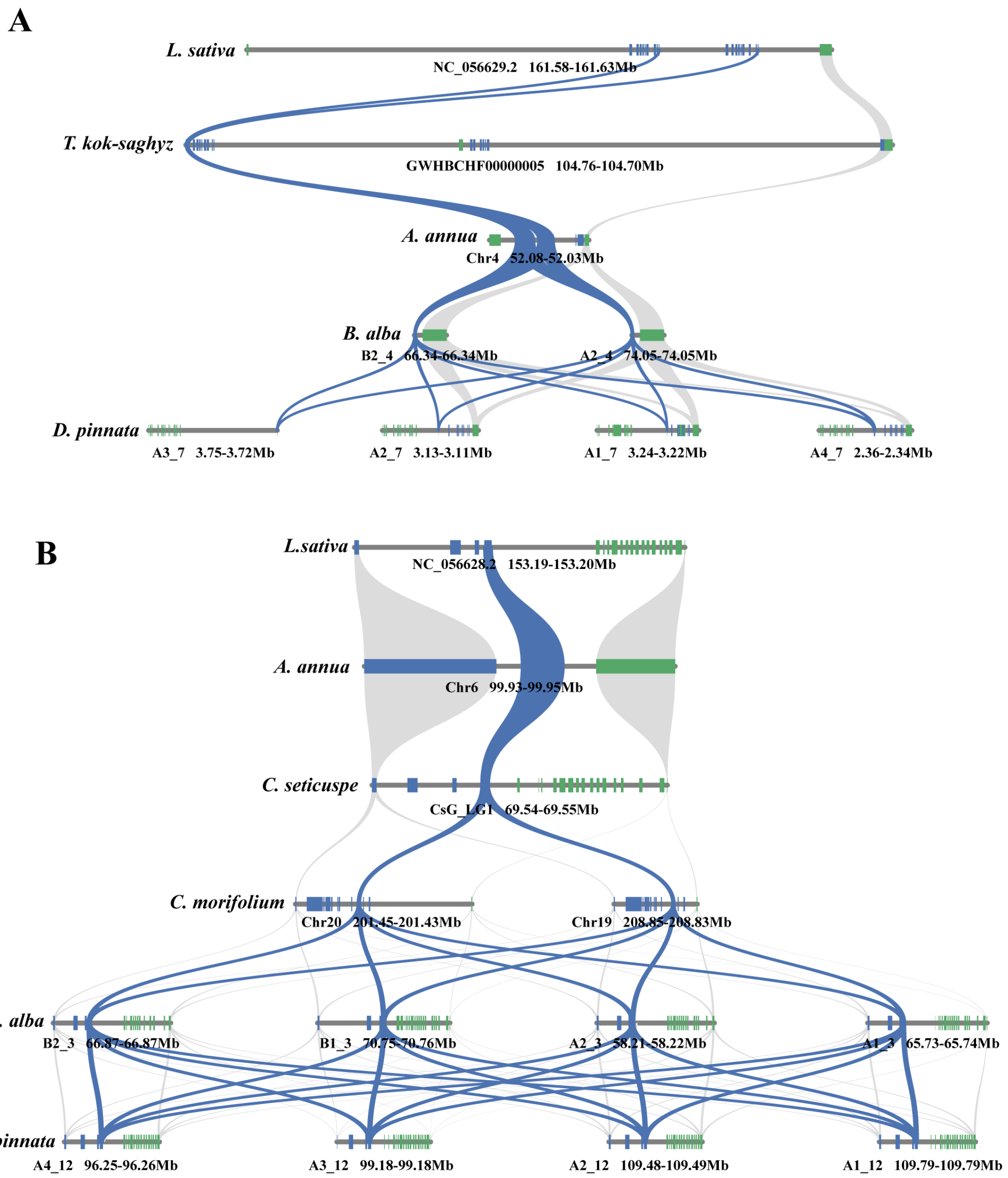


**Supplementary Figure 18**. Synteny relationships of the *HDR* and *MCS* genes. (A) Synteny relationships of the *HDR* gene among five Asteraceae species (*L. sativa, B. alba, D. pinnata, A. annua, T. kok-saghyz*). The lines connecting the syntenic *HDR* genes are highlighted in blue. (B) Synteny relationships of the *MCS* gene among six Asteraceae species (*L. sativa, A. annua, C. morifoliu, D. a pinnata, B. alba, C. seticuspe*). The lines connecting the syntenic *MCS* genes are highlighted in blue. Rectangles represent annotated genes, with genes on the reverse strand indicated in green and those on the same strand in blue. The lines connecting the syntenic genes are highlighted in blue. The grey lines represent the gene collinearity among the candidate species.

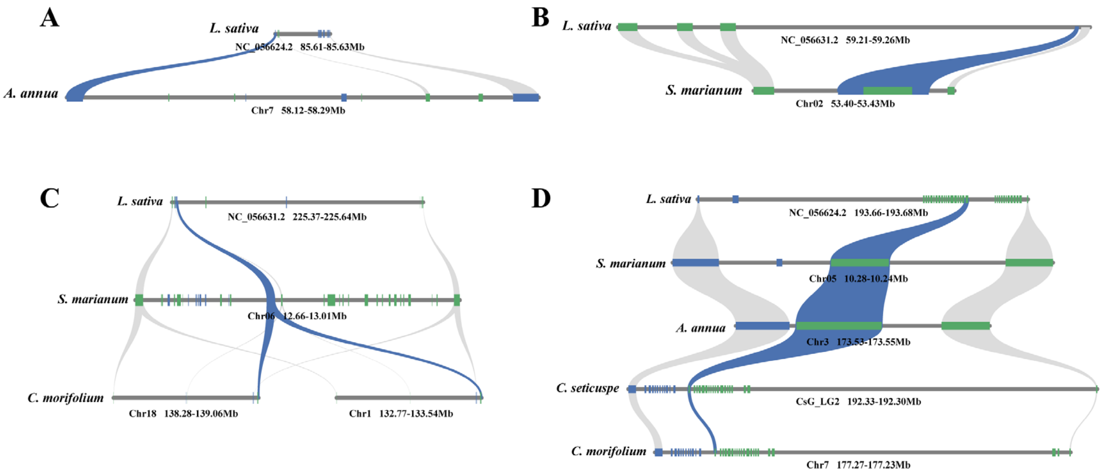


**Supplementary Figure 19**. Synteny relationships of the *CMK*, *MPD*, *CMS*, and *HDS* genes. (A) Synteny relationship of the *CMK* gene between *L. sativa* and *A. annua*. The lines connecting the syntenic *CMK* genes are highlighted in blue. (B) Synteny relationship of the *MPD* gene between *L. sativa* and *S. marianum*. The lines connecting the syntenic *MPD* genes are highlighted in blue. (C) Synteny relationships of the *CMS* gene among *L. sativa*, *S. marianum* and *C. morifolium*. The lines connecting the syntenic *CMS* genes are highlighted in blue. (D) Synteny relationships of the *HDS* gene among *L. sativa*, *S. marianum*, *A. annua*, *C. seticuspe* and *C. morifolium*. The lines connecting the syntenic *HDS* genes are highlighted in blue. Rectangles represent annotated genes, with genes on the reverse strand indicated in green and those on the same strand in blue. The lines connecting the syntenic genes are highlighted in blue. The grey lines represent the gene collinearity among the candidate species.

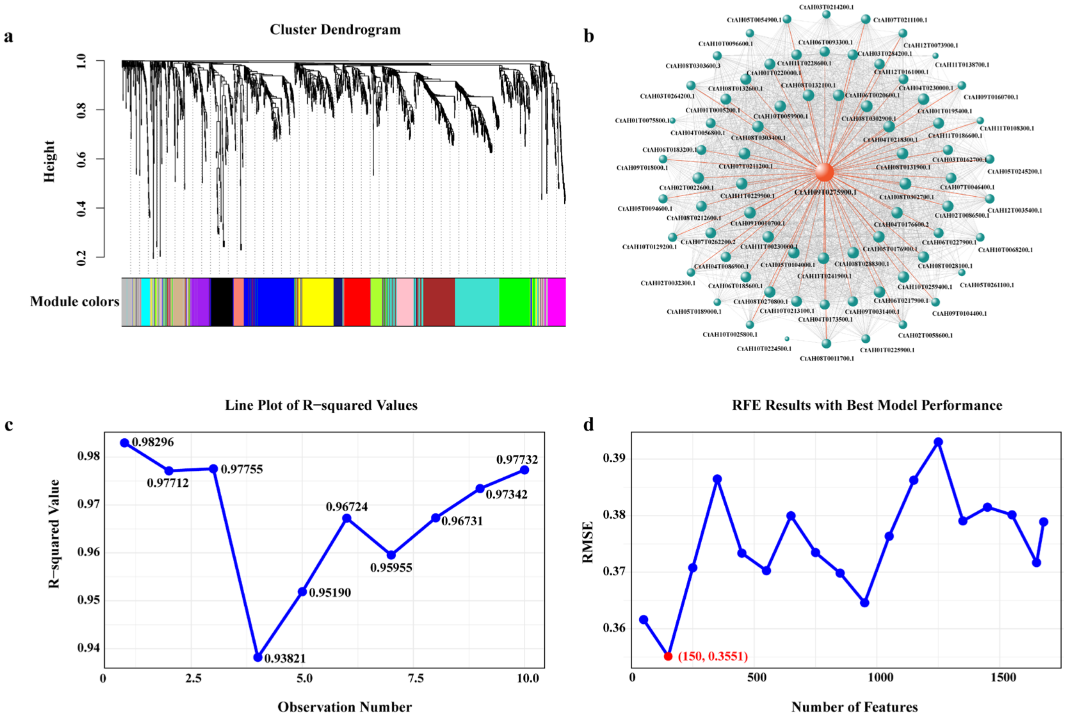


**Supplementary Figure 20**. WGCNA module characteristics and machine learning models' R-squared ($R^2$) and Root Mean Squared Error (RMSE) Performance (A) Clustering dendrogram of WGCNA. (B) Transcription factors co-expressed with *CtTPS* in the magenta module. (C) The $R^2$of ten independent random forest models. (D). Performance results of the SVM-RFE model: The curve depicts the changes in RMSE with the number of features, where the red dot marks the optimal performance point (150 features, RMSE = 0.3551).

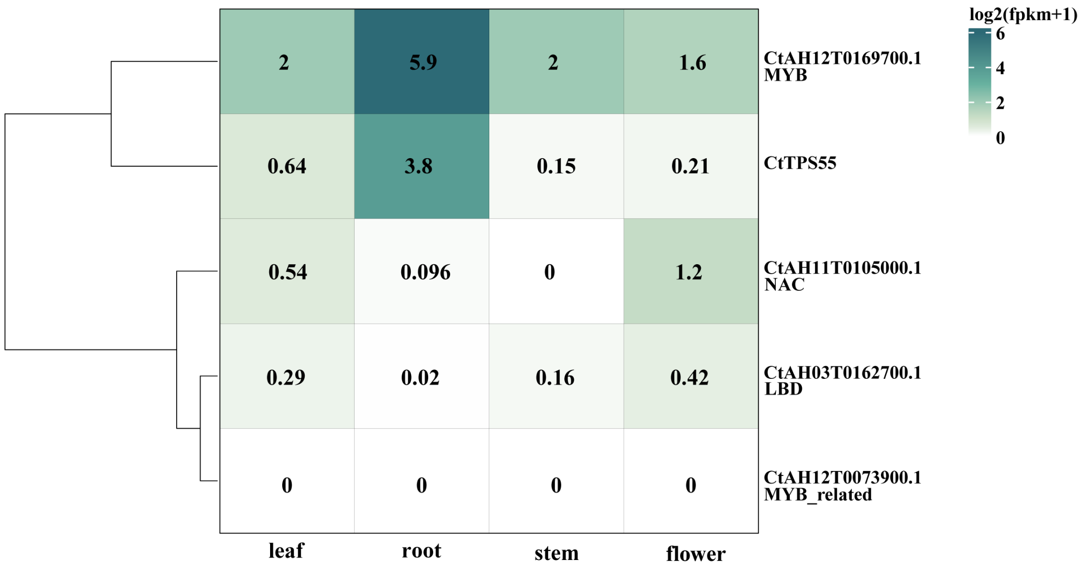


**Supplementary Figure 21**. Expression of four key transcription factors obtained by screening *CtTPS55* in roots, stems, leaves and flowers of safflower. The transcriptome data were from NCBI Sequence Read Archive under the accession number SRP411575.

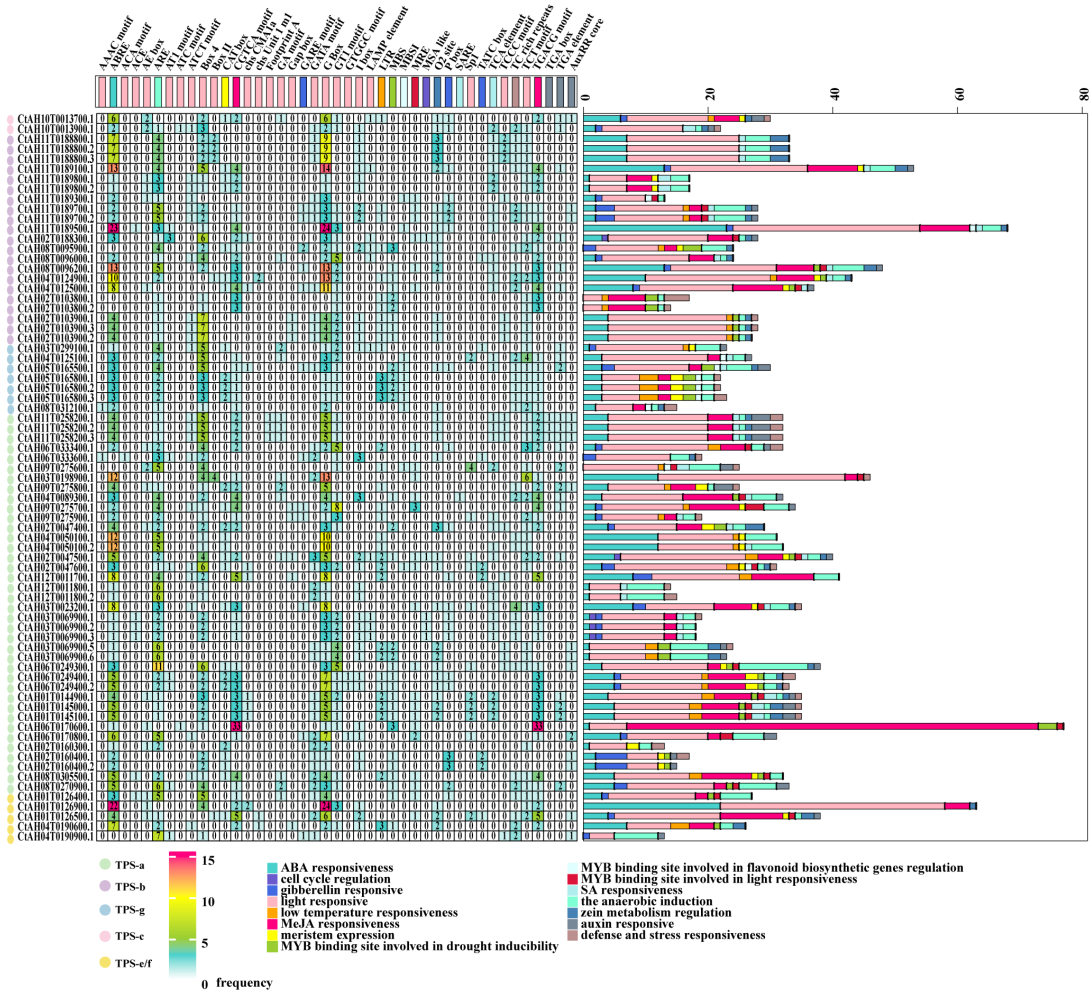

**Supplementary Figure 22**. Composition of cis-acting elements in the 2,000 bp region upstream of the *C. tinctorius* TPS genes.

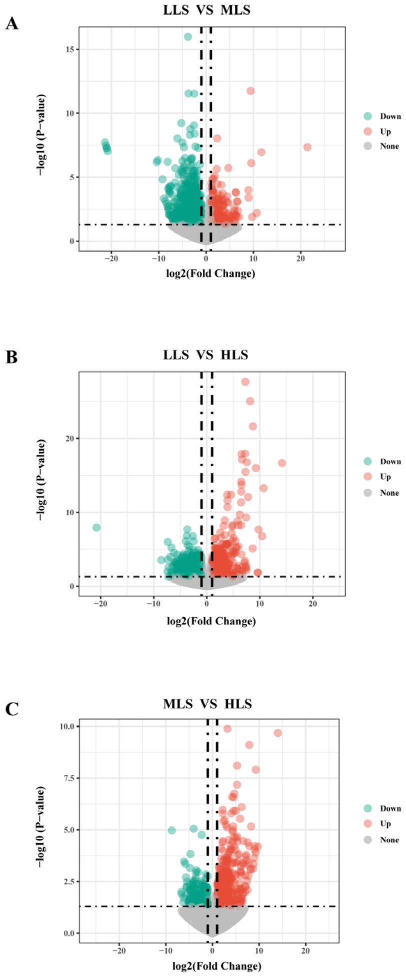


**Supplementary Figure 23**. Identification of differentially expressed genes in safflower under varying light intensity conditions. (A) Differential expression analysis of transcriptome samples under low-intensity light (LLS) and medium-intensity light (MLS). (B) Differential expression analysis of transcriptome samples under low-intensity light and high-intensity light (HLS). (C) Differential expression analysis of transcriptome samples under medium-intensity light and high-intensity light. Red scatters indicate genes that are significantly up-regulated under high-intensity light in the comparison group, green scatters indicate genes that are significantly down-regulated, and gray scatters are genes that are with non-significant expression differences.

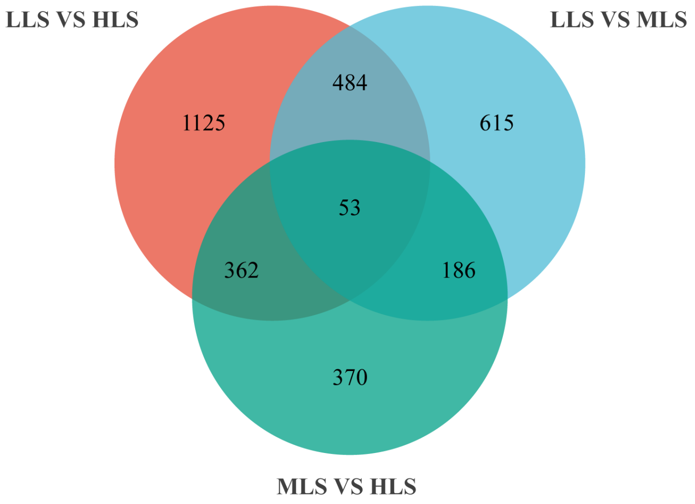


**Supplement Figure 24**. Venn diagram of differentially expressed genes under three intensities of light.

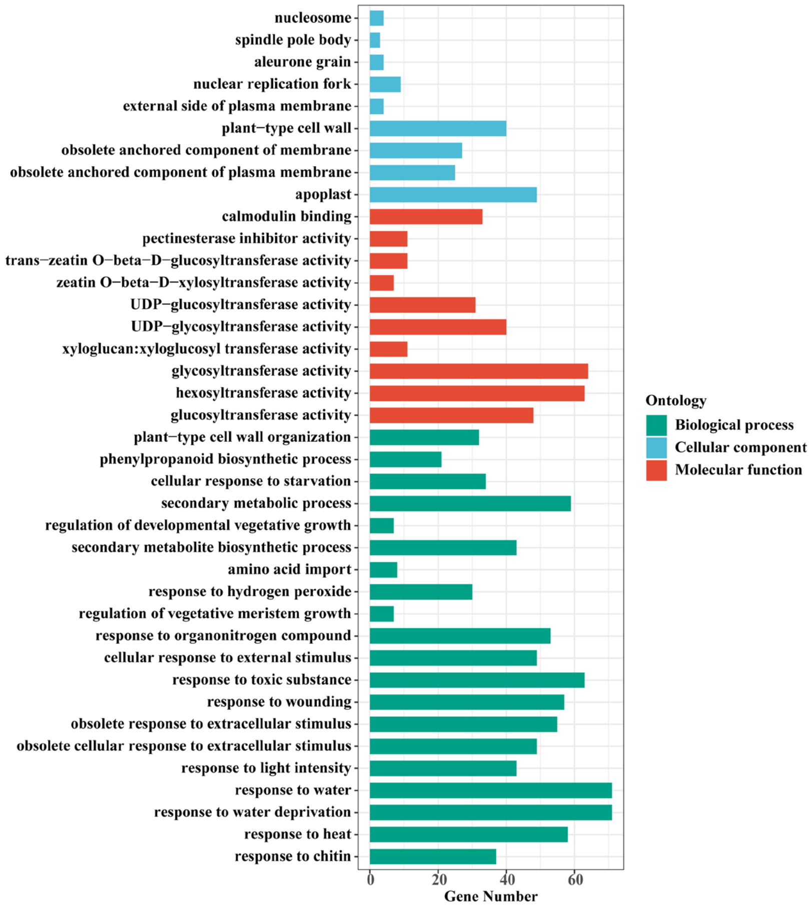


**Supplementary Figure 25**. GO enrichment analysis of differentially expressed genes.

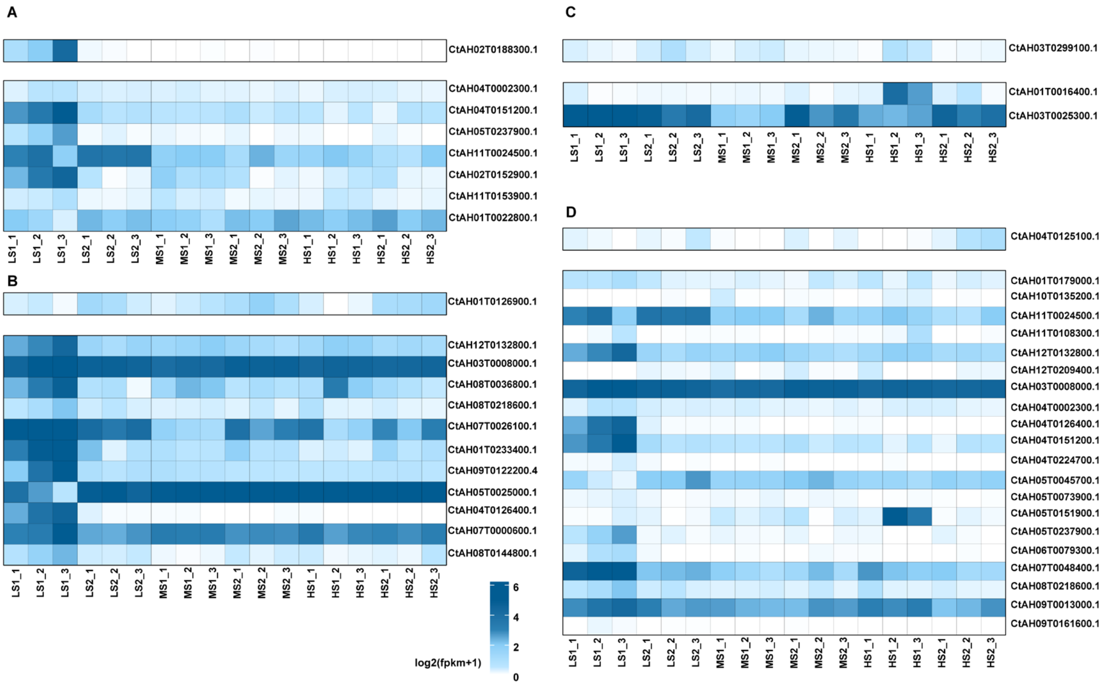


**Supplementary Figure 26**. Transcriptome analysis of transcription factors (TFs) and *CtTPS* under varying light intensities and in different tissues. (A) Expression levels of gene CtAH02T0188300.1 and seven TFs under different light intensities and across various tissues. (B) Expression levels of gene CtAH01T0126900.1 and eleven TFs under different light intensities and across various tissues. (C) Expression levels of gene CtAH03T0299100.1 and two TFs under different light intensities and across various tissues. (D) Expression levels of gene CtAH04T0125100.1 and twenty TFs under different light intensities and across various tissues. LS: low-intensity light, MS: medium-intensity light, HS: high-intensity light, I: first stage, II: second stage.
